# Delayed correlations improve the reconstruction of the brain connectome

**DOI:** 10.1371/journal.pone.0228334

**Published:** 2020-02-19

**Authors:** Mite Mijalkov, Joana B. Pereira, Giovanni Volpe

**Affiliations:** 1 Department of Neurobiology, Care Sciences and Society, Karolinska Institutet, Stockholm, Sweden; 2 Department of Physics, University of Gothenburg, Gothenburg, Sweden; Universidade de Lisboa, PORTUGAL

## Abstract

The brain works as a large-scale complex network, known as the connectome. The strength of the connections between two brain regions in the connectome is commonly estimated by calculating the correlations between their patterns of activation. This approach relies on the assumption that the activation of connected regions occurs together and at the same time. However, there are delays between the activation of connected regions due to excitatory and inhibitory connections. Here, we propose a method to harvest this additional information and reconstruct the structural brain connectome using delayed correlations. This delayed-correlation method correctly identifies 70% to 80% of connections of simulated brain networks, compared to only 5% to 25% of connections detected by the standard methods; this result is robust against changes in the network parameters (small-worldness, excitatory vs. inhibitory connection ratio, weight distribution) and network activation dynamics. The delayed-correlation method predicts more accurately both the global network properties (characteristic path length, global efficiency, clustering coefficient, transitivity) and the nodal network properties (nodal degree, nodal clustering, nodal global efficiency), particularly at lower network densities. We obtain similar results in networks derived from animal and human data. These results suggest that the use of delayed correlations improves the reconstruction of the structural brain connectome and open new possibilities for the analysis of the brain connectome, as well as for other types of networks.

## Introduction

The brain is a complex network whose structure consists of neurons and their anatomical connections, known as the connectome [1]. The connectome shapes the functional interactions between brain regions [2–4], which in turn are closely associated with behavior and cognitive functions [5–7]. Structural and functional brain connectivity differ in many fundamental aspects: structural connectivity is determined by the white matter fibers or axonal projections [8, 9], while functional connectivity describes the statistical dependencies in the activation signals between brain regions [10, 11]. Although many studies have shown similarities in the topography of structural and functional connections in the brain [12–16], their exact relationship remains unclear [7, 17]. For example, while the presence of an anatomical connection between two brain areas is associated with a stronger functional connection between them, functional connections are also present between brain areas without direct anatomical connections [15, 16].

There are several different non-invasive techniques that can assess structural and functional brain connectivity. Structural brain connectivity is often measured as the integrity of white matter fibers with diffusion imaging [18, 19]. However, the success of this approach is currently limited by the identification of false fibers and the suboptimal coverage of fibers with complex geometry [20, 21]. Functional brain connectivity is typically assessed from the correlations between the activation time series of brain regions obtained using functional magnetic resonance imaging (fMRI), electro-encephalography (EEG), or magneto-encephalography (MEG) [22–24]. This approach also suffers from an important drawback: it considers brain activity as a static phenomenon despite ample evidence that it is a dynamic process that changes over time [14, 25, 26]. In particular, it does not account for the fact that activation signals are typically generated in one brain region and then propagated to other ones [27], which entails causality and delays in the activation of various brain regions. Therefore, capturing and using the information stored in this complex temporal delay framework is necessary to achieve a coherent characterization of the functional connectivity [28–32].

In particular, here we show that the temporal delay in activation signals between two brain regions can be used to predict the relative strength of their structural connection. This novel method, the delayed-correlation, is able to predict 70% to 80% of connections of a structural network from its activation time series, overcoming the performance of other conventionally used same-time functional connectivity methods in predicting structural connectivity. Moreover, this method predicts better several regional structural network properties, indicating it provides a more accurate description of a brain network at a finer scale. Finally, it also predicts well the structural connections of the mouse, cat, macaque and human connectomes. Thus, this novel approach can provide a better understanding of the relationship between structural and functional connectivity, predicting how changes in brain network structure potentially give rise to abnormal functional dynamics in neurodegenerative and psychiatric diseases.

## Results

### Reconstruction of the brain connectome using delayed correlations

Temporal delays between the activations time series can arise, for example, due to the spatial distribution of brain regions and the finite transmission speeds between them [33, 34]. Brain regions that are more closely connected to each other are expected to activate with a much shorter delay than regions that are more loosely connected [34, 35]. Building on this observation, we propose to measure the delay *d*_max_, at which the activations of couples of brain regions are maximally correlated and, then, to use the inverse delay $d_{\text{max}}^{-1}$ to define their connection strength (Methods “Delayed-correlation method”).

To highlight the role of the temporal delays in the prediction of the structural connections, we compare our results against the corresponding same-time approaches most commonly employed to reconstruct brain connectivity from brain activation signals. These methods determine the connectivity strength between two nodes by calculating the same-time correlation coefficient between their activation time series or electrical activity [36]. This correlation coefficient can be either positive or negative. Since most graph theoretical tools do not deal with negative correlation coefficients, these are either substituted by their absolute values (*absolute correlation method*) or set to zero (*same-time correlation method*) (Methods “Absolute and same-time correlation methods”).

To compare the performance of these three methods, we simulate the activation of networks with a small-world organization [37], which has been shown to be essential for healthy brain function [38] and consistently observed in a wide range of human networks obtained by various imaging modalities [39]. Our analysis is focused primarily on low-density sparse networks because they match better biological networks, maximizing the balance between increased efficiency and lower cost [39, 40]. The strength of the connections between the nodes is defined using a symmetric q-Gaussian distribution, whose parameter *q* can be adjusted to test different distributions of connection strengths, from Gaussian (*q* = 1) to heavy-tailed distributions (*q* > 1) (Methods “Construction of simulated networks”). We simulate the spontaneous functional neuronal activity in each node using a linearized Wilson-Cowan dynamics model, which has been widely used to assess the relationship between structural and functional brain connectivity [15, 41] (Methods “Network dynamics”).

**Fig 1:**
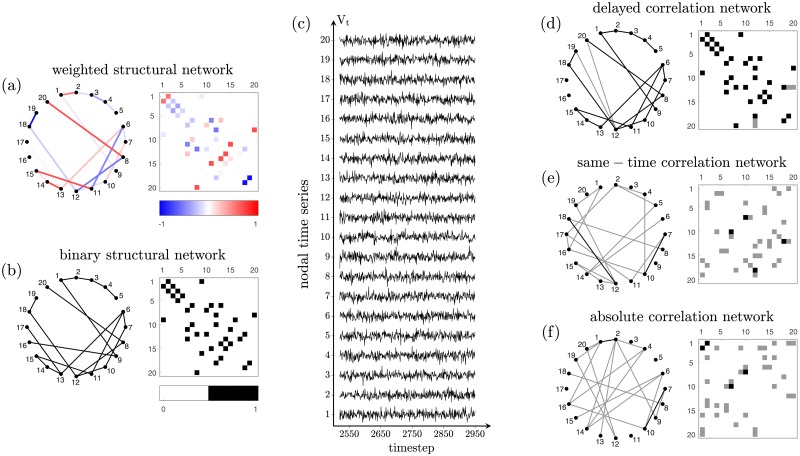
Network reconstruction procedure. (a) Example of a weighted small-world structural network and (b) corresponding binary network. In this figure, for illustration purposes, we show a small network of 20 nodes, but we use networks of 200 nodes in the rest of this study. (c) Examples of activation time series for each node, simulated by implementing a linearized Wilson-Cowan dynamics. (d-f) Reconstruction of the structural network from the information contained in these time series, using (d) the delayed-correlation method (retaining the connections with shortest delay), (e) the absolute correlation method (retaining the connections with largest absolute Pearson’s correlation coefficients), and (f) the same-time correlation method (retaining the connections with largest Pearson’s correlation coefficients); the edges that are correctly reconstructed are shown in black, and the edges that are incorrectly reconstructed are shown in gray (compare with the original network shown in (b)).

Fig 1 demonstrates the procedure for network reconstruction on a network with only 20 nodes, for illustration purposes. The network to be analyzed is shown in Fig 1a together with its weighted connectivity matrix. The corresponding binary network and matrix are shown in Fig 1b. Simulating the activation time series of each node, we obtain the activation time series shown in Fig 1c. Finally, we apply the different methods to reconstruct the structural network from the activation time series. By comparing Fig 1d–1f, it is clear that the delayed-correlation method performs much better than the absolute and same-time correlation methods in predicting the underlying structural connectivity. In all cases, the weighted networks obtained from these methods are binarized by retaining only the connections with largest inverse delays or with the highest correlation coefficients in order to reach the desired density. In Fig 1d we can see that the network reconstructed using the delayed-correlation method overlaps well with the underlying binary structural network shown in Fig 1b. In contrast, only two edges from the networks reconstructed using the absolute correlation method (Fig 1e) and the same-time correlation method (Fig 1f) are also present in the structural network shown in Fig 1b. These differences in overlap become even clearer by focusing on a set of nodes such as those in the path 1 − 2 − 3 − 4 − 5 of Fig 1b. The delayed-correlation method correctly identifies this sequence (Fig 1d), whereas the absolute and same-time correlation methods introduce a false connection between nodes 2–4 and miss the connections between nodes 2–3 and 3–4 (Fig 1e and 1f).

### Accuracy of network reconstruction with different methods

**Fig 2:**
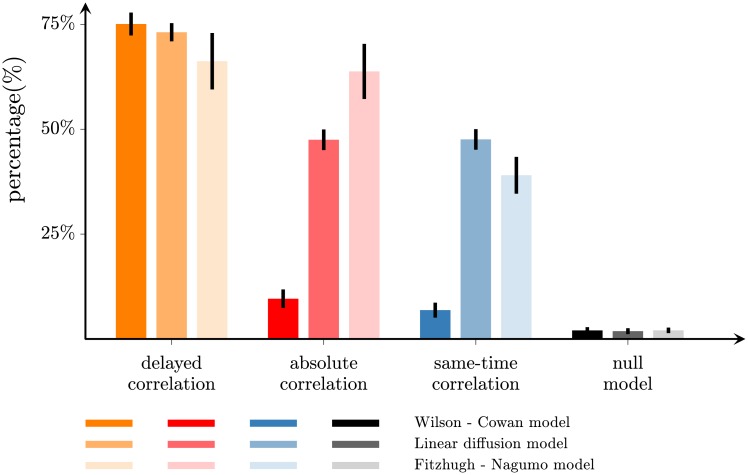
Accuracy of network reconstruction. Percentage of edges in the structural network correctly identified by the delayed-correlation method (orange), the absolute-correlation method (red), and the same-time correlation method (blue) for a 200-node network thresholded at 2% density. The black bars represent the null model (random network). The network activation dynamics was simulated with linearized Wilson-Cowan (leftmost bars), diffusion (middle bars), and Fitzhugh-Nagumo (rightmost bars) models. The error bars represent the standard deviation over 100 trials. The structural networks are small-world networks with *β* = 0.05, and the connection strengths are drawn from a symmetric q-Gaussian distribution with *q* = 1.

The percentage of connections that are correctly reconstructed by the different methods are shown in Fig 2 (leftmost bars for Wilson-Cowan dynamics) for networks of 200 nodes thresholded at 2% density. The delayed-correlation method correctly identifies 75 ± 3% of the connections. In contrast, the absolute and same-time correlation methods identify only 9.6 ± 2.2% and 6.9 ± 1.8% of the connections, respectively. We also compare these results to a null model where connections are random (*random method*) in order to assess whether the obtained results can be explained by chance. The null model identifies correctly only 2.0 ± 0.8% of the connections. Therefore, the performance of the delayed-correlation method is considerably better than the null model, while the performance of the other two methods is only marginally better i.e. the difference is approximately 70% for the delayed correlation method compared with 7% and 5% for the absolute and same-time correlation, respectively. We have verified that similar results are also obtained for different network sizes (100 and 500 nodes) and for other densities (up to 14%), as shown in Table A in S1 Appendix.

Additionally, the absolute and same-time correlation methods often mistake indirect connections for direct ones, as shown in Table B in S1 Appendix. For example, in Fig 1e and 1f, they identify a connection between nodes 2–4, which are only indirectly connected through node 3. This agrees with previous studies that have suggested that indirect structural connections can produce strong correlated activation in regions that lack a direct anatomical link [15].

### Accuracy of network reconstruction with different nodal activation dynamics

The results shown above are consistent with those obtained using alternative models to simulate the network dynamics in addition to a linearized Wilson-Cowan model [42] (Fig 2, leftmost bars). First, we use a model that estimates the network dynamics as a diffusion process over the structural network (Methods “Linear diffusion model”) [43]. The patterns generated by this model have been shown to match those empirically observed in functional connectivity better than other linear and non-linear models [43]. Also in this case, the delayed-correlation method is able to correctly reconstruct 73 ± 2% of the connections, compared to only 48 ± 3% and 48 ± 2% of the connections reconstructed by the absolute and same-time correlation methods (Fig 2, middle bars and Table C in S1 Appendix). Second, we use the Fitzhugh-Nagumo model of spiking neurons (Methods “Fitzhugh-Nagumo model”) [44, 45]. This model has been shown to capture the dynamic behavior of large-scale, biologically-based neuronal networks [46]. To emphasize the origin of the temporal delays due to the network interactions, we couple the Fitzhugh-Nagumo oscillators with linear and instantaneous terms, in contrast to previous studies that explicitly included temporal delays in the model [35]. The delayed-correlation method correctly reconstructs 66 ± 7% of the connections, outperforming the absolute and same-time correlation methods (Fig 2, rightmost bars and Table D in S1 Appendix).

As shown in Fig 2, the delayed correlation method shows a striking increase in performance when compared to the same-time approaches for the Wilson-Cowan model; however this difference is less pronounced in the diffusion and Fitzhugh-Nagumo models. In particular, the absolute and same-time correlation methods show increased performance for these models which can be due to the fact that the functional networks derived by these models include contributions from both instantaneous and delayed nodal activations. In the Fitzhugh-Nagumo model, this arises as a consequence of the existence of relatively broad spikes in the nodal time series, which, when combined with the use of long time windows to derive the functional connectivity, lead to the inconclusive separation between the coactivation and delayed activation signals between the network nodes [47]. Similarly, the diffusion model captures best only the stationary correlation structure of the functional connectivity without conveying any information about distance and path delays between the network nodes [43]. The effect of path delays only becomes evident after introducing noise in all nodes’ dynamics and driving the system out of equilibrium at each simulation step, therefore inducing an oscillatory behavior into the model.

### Measurement of global and nodal network measures

**Fig 3:**
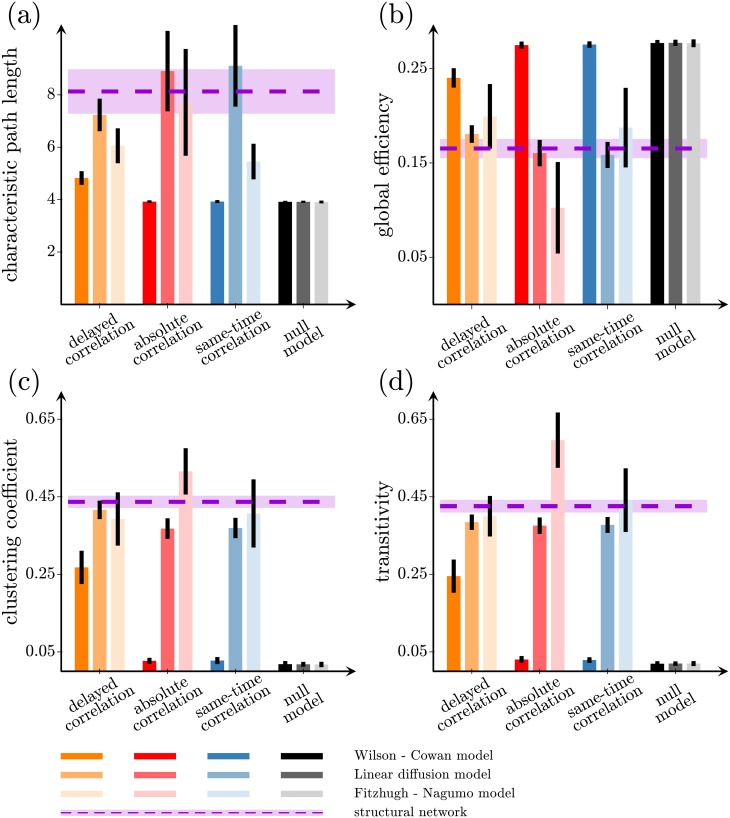
Global network measures from reconstructed networks. (a) Characteristic path length, (b) global efficiency, (c) clustering coefficient, and (d) transitivity of the underlying structural network (dashed violet line), and of the networks reconstructed using the delayed-correlation method (orange bars), the absolute-correlation method (red bars), the same-time correlation method (blue bars), and the null model (black bars). The network activation dynamics was simulated with linearized Wilson-Cowan (leftmost bars), diffusion (middle bars), and Fitzhugh-Nagumo (rightmost bars) models. Each bar denotes the average of 100 simulations; the error bars represent one standard deviation. The 200-node networks are as in Fig 2.

To quantitatively assess the ability of these methods to measure the global network topology, we computed the following global network measures in the reconstructed networks and compared them to those of the underlying network: characteristic path length (Fig 3a), global efficiency (Fig 3b), clustering coefficient (Fig 3c) and transitivity (Fig 3d) (Methods “Definition of the graph measures”). Since the structural networks we are considering have a small-world architecture, they feature short path lengths, high global efficiency, high clustering, and high transitivity. The values of these measures in the structural networks are shown by the violet dashed lines in Fig 3. For the Wilson-Cowan model, the absolute correlation and same-time correlation methods perform similarly to the null model for all global measures, while the delayed-correlation method is the only one to perform better, especially for the clustering coefficient (Fig 3c, leftmost bars) and transitivity (Fig 3d, leftmost bars). We obtain similar results for different network sizes and densities, as shown in Table E in S1 Appendix. Fig 3(middle and rightmost bars) indicate that delayed correlation retains good performance also for diffusion and Fitzhugh-Nagumo model respectively. Incidentally, the performance of the absolute and same-time correlation methods increase for both models when compared to Wilson-Cowan model, as a consequence of their ability to identify larger number of correct structural connections as discussed above.

**Fig 4:**
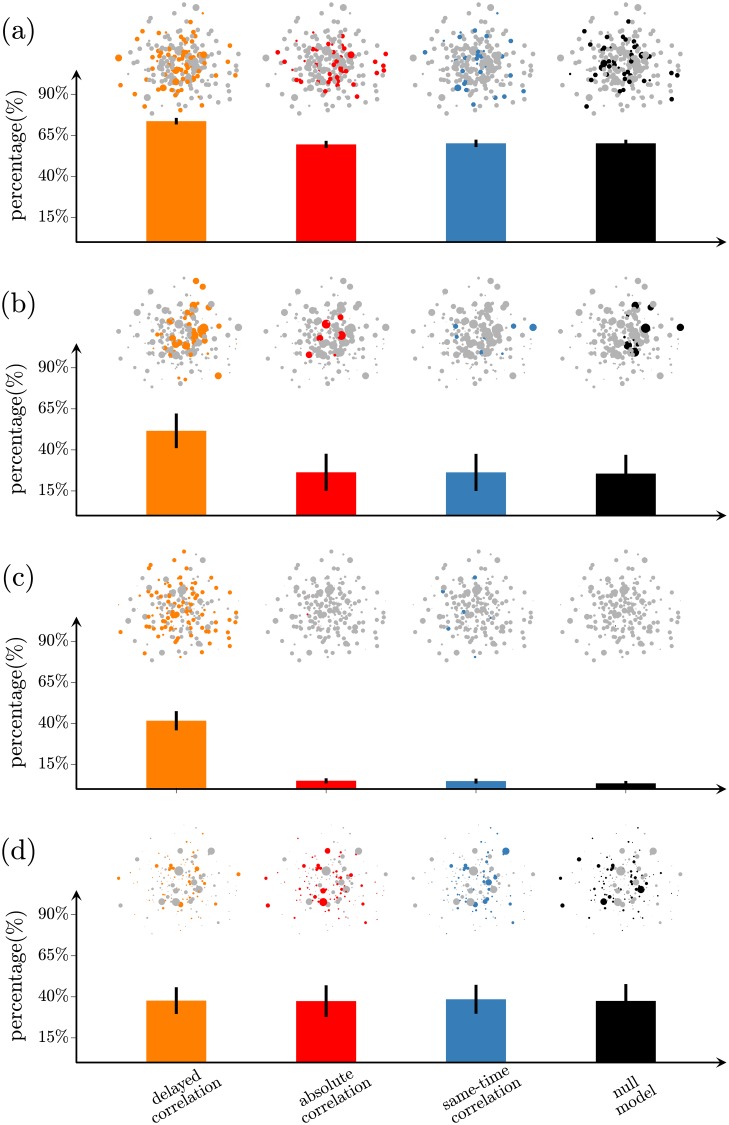
Nodal network measures in the reconstructed networks. (a-d) Average percentage of correctly determined nodal measures: (a) nodal degree, (b) nodal global efficiency, (c) nodal clustering and (d) eigenvector centrality from the networks reconstructed using the delayed-correlation method (orange), the absolute correlation method (red), the same-time correlation method (blue), and the null model (black). In the insets, the size of the symbols represent the values of the measures in the real network, while the colored symbols represent the nodes whose nodal measures have been correctly determined.

We also assess the reconstruction of network topology at the local level by computing the nodal degree (Fig 4a), nodal global efficiency (Fig 4b), nodal clustering coefficient (Fig 4c) and eigenvector centrality (Fig 4d). For networks with 2% density, the delayed-correlation method identifies the nodal degree with 74 ± 2% accuracy, the nodal global efficiency with 52 ± 11% accuracy, the nodal clustering coefficient with 42 ± 6% accuracy and the eigenvector centrality with 38 ± 8%. These accuracies are considerably better than those achieved by the absolute correlation method (59 ± 2%, 26 ± 11%, 5.0 ± 1.5% and 37 ± 10% for the first three measures) and the same-time correlation method (60 ± 2%, 26 ± 11%, 4.8 ± 1.5% and 39 ± 9%), which are in fact close to the performance achieved by the null model (60 ± 2%, 26 ± 11%, 3.5 ± 1.4% and 38 ± 10%). These findings show that the two later methods show comparable performance as random chance. Similar results are also obtained for higher densities (Table F in S1 Appendix), although all methods decrease their performance.

As Fig 4a–4d shows, the delayed-correlation method’s accuracy is highest for the nodal degree, decreases for the nodal global efficiency and clustering coefficient, and it is lowest for the eigenvector centrality. These results can be explained by considering how the various measures describe the different levels of influence the node has within the network. Specifically, the nodal degree characterizes the influence a node has only on its neighbors, and therefore its measurement requires only the correct reconstruction of single edges. This is not the case for the nodal global efficiency, which requires the simultaneous reconstruction of multiple edges to fully reconstruct the shortest paths of various lengths. Additionally, the nodal clustering coefficient requires triplets of edges to be correctly reconstructed at the same time, while the eigenvector centrality requires even more accurate network-wide reconstruction. Therefore, the performance of the delayed-correlation method for different measures decreases with the higher network-wide influence those measures describe, and demonstrates that the delayed-correlation method is particularly effective in reconstructing network measures that convey the local interactions of a given node.

Furthermore, we plot the degree distributions identified by the different correlation methods for regular, small-world and random networks (Fig Aa, Fig Ab and Fig Ac in S1 Appendix respectively). The distributions were calculated for networks of 200 nodes thresholded at 2% and were averaged over 100 trials. All nodes have an equal degree in a regular network (for networks of 200 nodes thresholded at 2% the degree is 4, as shown by the violet bars in Fig Aa in S1 Appendix), and only few nodes change their degrees in a small-world network due to the random rewiring of a small number of edges (Fig Ab in S1 Appendix, violet bars). Regular and small-world networks reconstructed by the delayed-correlation method (Fig Aa and Ab in S1 Appendix, orange bars) have a symmetric degree distribution centered around the most common degree value for the structural network. Therefore, a large fraction of the network nodes retain their degrees, as a result of the fact that most of the structural connections are correctly reconstructed (see also Fig 2). The changes in the nodal degrees are a direct consequence of the wrongly reconstructed connections. This indicates that a large number of the wrongly reconstructed connections are rewired as indirect edges between 2^nd^–3^rd^ neighbors, in agreement with the results presented in Table B in S1 Appendix. In all cases, the degree distributions of the absolute and the same-time correlation methods are identical to those of the null model, again indicating that they do not perform better than change. Moreover, as expected for random structural networks (Fig Ac in S1 Appendix), the degree distributions of all models become similar.

### Robustness of the delayed-correlation method

**Fig 5:**
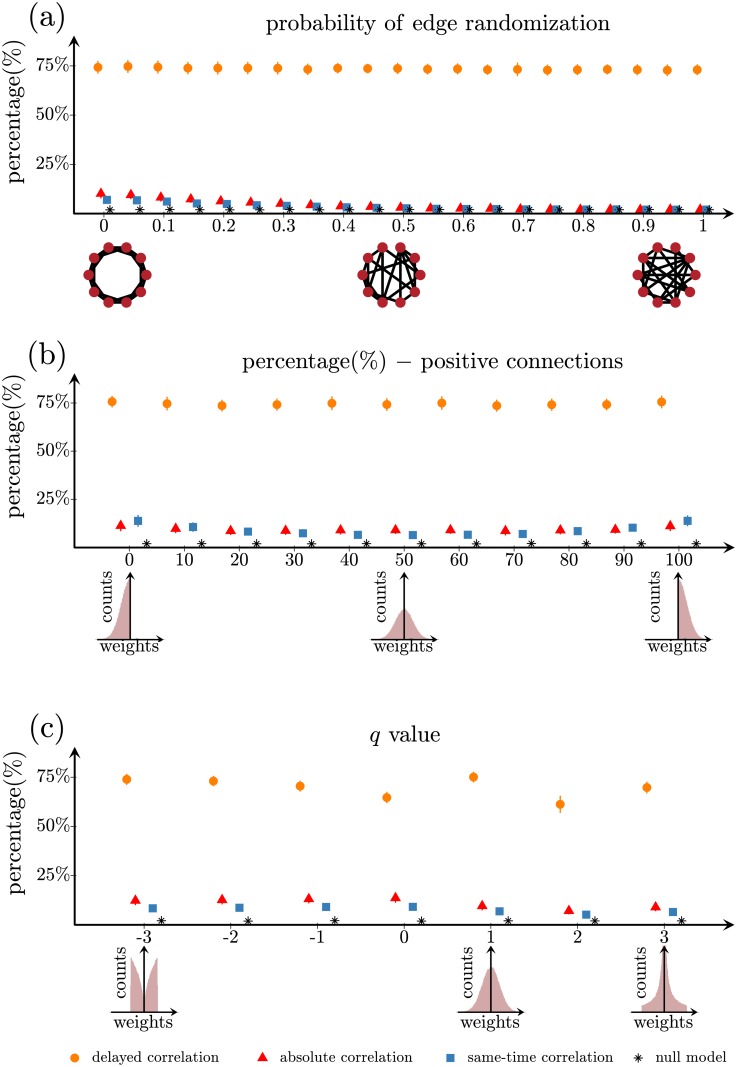
Performance of the delayed-correlation method for different types of networks. Reconstruction efficiency as a function of (a) probability to randomize an edge in the Watts-Strogatz model, (b) percentage of positive weights in the weighted structural network, and (c) different distribution of weights in the structural network represented by the *q* parameter in a q-Gaussian distribution. The error bars represent two standard deviations. Insets: (a) Examples of a structural network for different randomization parameters in the Watts-Strogatz model; the networks vary from regular (left) to random networks (right). (b) Histograms of the structural weights distribution from 0% (left) to 50% (middle) and 100% (right) positive weights. (c) Weight distribution changes from bounded q-Gaussian for *q* = −3, Gaussian function for *q* = + 1 to heavy tail distribution for *q* = + 3. In all cases, the structural network of 200 nodes is thresholded at 2% density and the results are averaged over 100 trials. The network activation dynamics was simulated with the linearized Wilson-Cowan model.

The delayed-correlation method works well for a broad range of network architectures and parameters. First, Fig 5a shows that it consistently predicts more than 73% correct connections in networks with different architectures, from regular to small-world, and from small-world to random. Second, Fig 5b shows that it is robust to variations in the ratio between excitatory and inhibitory connections. Finally, Fig 5c shows that changing the distribution of nodal connectivity strengths from bounded (for *q* = −3) to normal (*q* = 1) to heavy tailed (for *q* = 3) does not affect its performance. In all cases, the results obtained by the delayed-correlation method are better than those obtained by the absolute correlation method, the same-time correlation method and the null model (see also Tables G-K in S1 Appendix for analogous results obtained with diffusion and FitzHugh-Nagumo model). Similar results can also be observed for different network sizes and densities, for networks with different community structure (see section “Reconstruction of network community structure” and Fig B in S1 Appendix), as well as for different noise levels in the dynamics model (Fig C and Tables L and M in S1 Appendix).

### Reconstruction of biological networks

**Fig 6:**
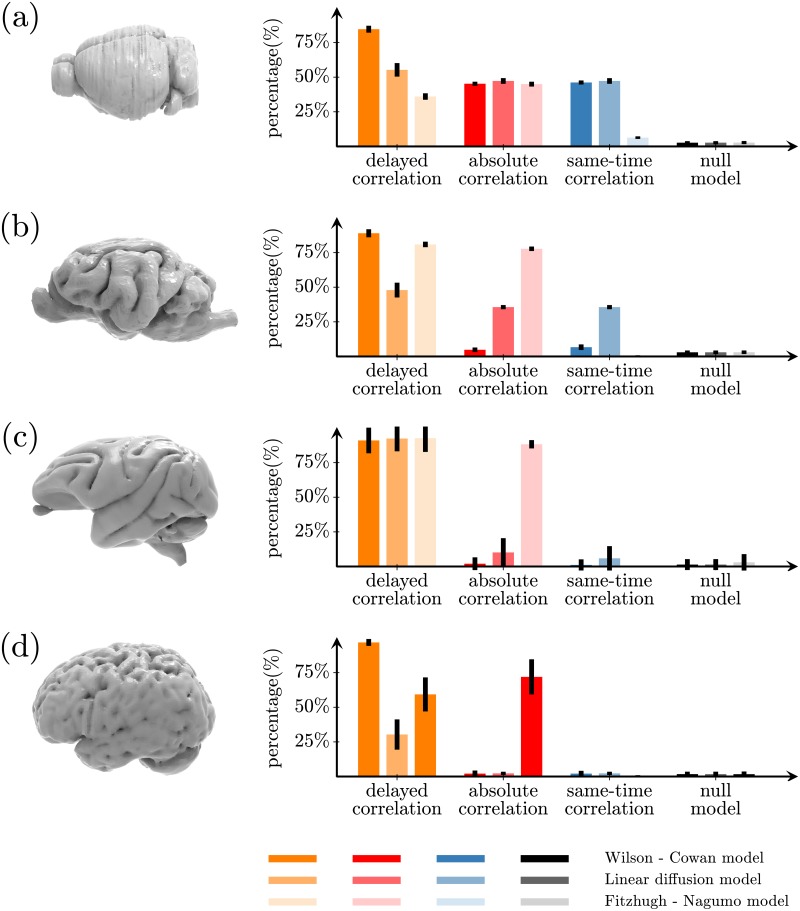
Reconstruction of biological networks. Percentage of edges in the structural network correctly identified by the delayed-correlation method (orange bars), the absolute-correlation method (red bars), the same-time correlation method (blue bars), and the null model (random network) for the (a) mouse, (b) cat, (c) macaque and (d) human connectomes thresholded at 2% density. In all cases, the network activation dynamics was simulated with linearized Wilson-Cowan (leftmost bars), diffusion (middle bars), and Fitzhugh-Nagumo (rightmost bars) models.

We further test whether these methods are able to reconstruct biologically meaningful networks such as the mouse [48, 49], cat [50], macaque [51, 52] and human [53] connectomes (Methods “Data for biological networks”). Similarly to previous analyses, we use a linearized Wilson-Cowan model to simulate functional network signals in the biological connectomes. Fig 6 (leftmost bars) shows the percentage of connections that can be identified for these connectomes after binarizing them at 2% density. The delayed-correlation method predicts a higher percentage of structural connections in the mouse (66.6% ± 1.6%, Fig 6a), cat (84.2% ± 2.2%, Fig 6b), macaque (90.5% ± 9.8%, Fig 6c) and human (96.8% ± 2.6%, Fig 6d) connectomes, compared to the absolute correlation (mouse: 38.7% ± 1.7%, cat: 3.9% ± 1.6%, macaque: 2.1% ± 5.0%, human: 2.0% ± 1.9%) and zero correlation (mouse: 39.8% ± 1.7%, cat: 5.3% ± 1.8%, macaque: 2.3% ± 4.8%, human: 2.1% ± 2.3%) methods. Similar results are also obtained for other densities (up to 14%), as shown in Table N in S1 Appendix. Analogous results for the case of simulation of functional signals with the diffusion and Fithzugh-Nagumo model are showed in Fig 6 by middle and right bars respectively as well as Tables O and P in S1 Appendix.

## Discussion

In this study, we propose a method to reconstruct the connections and topological properties of a structural network from its activation signals. By using the information contained in delayed temporal correlations, this method correctly identifies up to 80% of structural connections, and is able to determine global and nodal network measures. These results indicate that temporal delays play a crucial role in bridging the gap between functional and structural connectivity.

Computational models have become increasingly important to study the relationship between the structure of the brain connectome and its patterns of activation [54]. For example, several studies simulated the brain network dynamics using realistic anatomical structural networks [15, 55], other developed alternative dynamics models [43], which could then be used to predict topological properties of the structural network [41]. These studies found that functional connections exist also between regions with no direct structural connections [15, 56, 57]. However, they used same-time correlations to estimate the functional connections, which assume that brain regions get activated together and at the same time. Instead, the delayed interactions in a network have been incorporated in a variety of methods, for example multivariate autoregressive models [58, 59], Granger causality [60, 61], dynamic time-warping [62, 63] and maximal delay correlation [64], which were able to provide a better characterization of the functional connectivity. Building on the assumption that quasi-simultaneous brain activity can only occur between nodes connected by direct paths, our results add to this body of literature by suggesting that the temporal delays can be used to discriminate direct from indirect connections. The proposed method can predict up to 80% of the structural connections, thus offering a simple and intuitive picture that can explain the interactions between regions.

While the delayed-correlation method performs well for a set of different types of networks, it achieves its best performance for sparse networks, which are most commonly encountered in the human brain [9]. Sparse networks contain fewer, but stronger functional connections, which are more likely to be mediated by direct anatomical connections. In contrast, networks that are more densely connected contain weaker functional connections, which tend to be mediated through polysynaptic structural connections [13, 65]. These findings are confirmed by our observation that, as the density of the network increases, the functional connections overlap with an increasing number of indirect connections of various path lengths.

Previous studies have reported differences in the global topological organization between structural and functional networks [66]. However, despite these differences, there is evidence that the graph properties of structural networks can influence some of the features of their corresponding functional networks [14, 67]. Our results are in line with these observations. In global network topology, we observe a mismatch between the clustering coefficients of the structural and functional networks; however these networks share similar global efficiencies. At the nodal level, the majority of nodes in the delayed-correlation functional networks have similar nodal degrees, nodal clustering and global efficiency as in the structural networks. With the exception of the eigenvector centrality, these results are better than those obtained with the standard methods, again underlining the potential of temporal delays in capturing general network topology.

We also tested the robustness of the delayed-correlation method against variations of several network properties, including global network organization, ratio of excitatory-inhibitory connections, and distribution of inter-nodal strengths. Such variations can arise, for example, due to neurological disorders such as Alzheimer disease and autism [68–70]. Our results demonstrate that the emergence of temporal delays does not depend on the properties of the structural network and the particular model used to simulate its activity, indicating that the delayed-correlation method can be reliably applied to different networks, which may be present in different diseases.

Furthermore, we evaluated the performance of the delayed-correlation method with respect to different models of network activation dynamics. The spontaneous functional neuronal activity was simulated by employing linearized Wilson-Cowan, diffusion and FitzHugh-Nagumo models. The performance of the absolute and same-time correlation methods varied greatly between models, and in particular, was higher for the diffusion and FitzHugh-Nagumo models due to their tendency to include the contributions of the instantaneous coactivation between the network nodes [43, 47]. On the other hand, the delayed-correlation performed consistently better than the same-time activation methods and showed consistent behavior across models indicating the potential of this method to analyze a wide range of networks with different functional activation dynamics.

Finally, we also evaluated the ability of the delayed-correlation method to predict the underlying structural connections of the mouse, cat, macaque and human brain connectomes. Using animal connectomes provided by previous neural tracing studies and a population-averaged human connectome derived from a large sample of healthy individuals, we observe that the delayed-correlation method successfully identifies a much higher percentage of structural connections compared with the absolute and zero correlation methods. These results open new possibilities for the analysis of biological networks.

This study presents some limitations and opportunities for future work. We focused on demonstrating the underlying causes and general properties of the temporal delays in small neuronal networks and in the connectomes derived from neural tracing animal data or population-averaged human data. We do not make attempts to relate our observations to individual brain networks; in particular, the structural networks derived by diffusion imaging and functional activity between the brain regions obtained with fMRI in humans. This is due to strong evidence showing the caveats of diffusion imaging in resolving fiber trajectories for individual subjects, including the identification of false tracts and suboptimal coverage of small pathways with complex geometry [20, 21]. In fact, it has been previously shown that the percentage of valid white matter connections in individual subjects can vary between 3.75% to 92% when using diffusion tensor imaging [53, 71]. Additionally, in the case of fMRI, earlier studies demonstrated that various correlation metrics and preprocessing steps can result in very different functional networks [72], which may include spurious correlations due to motion that cannot be completely eliminated by preprocessing procedures [73]. In addition, we derive our results from stationary functional data (relatively long time series with 80000 samples), which permits us to interpret them using few topological measures of the structural matrix [41] and improves the structural-functional connection [14]; therefore it remains to be seen whether our observations will be affected by the dynamic properties of the functional connectivity at shorter time scales. The structural networks we study are topological, and therefore, we cannot assess whether our results fit with few measures that have been shown to have an effect on functional connectivity; for example, Euclidean distance between regions [74], short range vs. long range connections [75]. Despite these limitations, in this work we showed that the information stored in the temporal delays can be used to reconstruct functional networks that are highly predictive of the underlying structural networks. The temporal delays originate from the complex network-wide influence on the spontaneous dynamic activity, and as a result, could potentially offer a general framework to understand how structural architecture affects the functional interactions in health and disease.

## Methods

### Delayed-correlation method

The cross-correlation function between two discrete time series is their correlation as a function of their delay. This delay is the number of time steps by which one time series is shifted with respect to the other before calculating the correlation. Therefore, to calculate the strength of the functional connection between the nodes *j* and *k* with time series *x*_*j*_ and *y*_*k*_ of length *N* time steps, we calculate the biased cross-correlation function at a given delay *d* as

$$\begin{array}{c} r_{jk}\left(d\right)=\{\begin{array}{cc} \frac{1}{N}\sum_{n=0}^{N-d-1}x_{j}\left(n+d\right)x_{k}\left(n\right) & d\geq 0\\ r_{jk}\left(-d\right) & d<0 \end{array} \end{array}$$ (1)

and identify the delay *d*_max_ at which the absolute value of this function is maximal. Finally, the strength of the functional connection between nodes *j* and *k* is defined as 1/*d*_max_. The weighted functional network is obtained by repeating this calculation for all pairs of nodes; this network is subsequently binarized at the desired density.

### Absolute and same-time correlation methods

Using the standard absolute and same-time correlation methods, the functional connectivity between two nodes *j* and *k* with respective activation time series *x*_*j*_ and *x*_*k*_ is quantified by the Pearson’s linear correlation coefficient, calculated as

$$\begin{array}{c} r_{jk}=\frac{\text{cov}(x_{j},x_{k})}{\sigma_{j}\sigma_{k}}\;, \end{array}$$ (2)

where cov (*x*_*j*_, *y*_*k*_) represents the covariance of the time series and *σ*_*j*_ and *σ*_*k*_ are their respective standard deviations. The functional networks are built by calculating the Pearson’s coefficient between all pairs of nodes in the network. Finally, the negative correlation coefficients are either set to zero (same-time correlation method) or substituted with their absolute values (absolute correlation method).

### Construction of simulated networks

We simulated structural networks with a small-world organization using the Watts and Strogatz model [37], in which we start from a regular network and then randomly rewire each edge with a probability *β*_WS_. Small-world networks were obtained for small values of *β*_WS_.

The strength of the structural connections between the regions was derived from q-Gaussian distribution with probability density function given by

$$\begin{array}{c} PDF\left(x\right)=\frac{\sqrt{\beta }}{N_{q}}{\left[1+\left(1-q\right)x\right]}^{\frac{1}{1-q}}\left(-\beta x^{2}\right), \end{array}$$ (3)

where *N*_*q*_ is a normalization constant and *β* = 1 through all simulations. Depending on the value of *q*, this distribution can be varied between that of a bounded random variable and that of a heavy-tailed random variable. In particular, for *q* = 1, it recovers the probability density function of a Gaussian distribution.

We note that, even though the dynamics of the network is simulated on weighted structural networks, the small-world characteristics of the networks are evaluated on the corresponding binarized networks at all densities.

Unless otherwise stated, all structural networks are derived by setting *β*_WS_ = 0.05, the connection strengths are derived by setting *q* = 1, and the distribution of weights is centered at zero in order to ensure equal number of positive and negative weights.

### Network dynamics

To simulate the network dynamics, we use a linear model proposed by Galan [42]. It is a linearization of a Wilson-Cowan dynamics in the absence of external simulation, where the dynamics is driven only by uncorrelated Gaussian random noise, *η*(*t*). The discretized equation governing the dynamics is given by

$$\begin{array}{c} \vec{u}\left(t+\Delta t\right)=A\vec{u}\left(t\right)+\eta \left(t\right), \end{array}$$ (4)

where $\vec{u}$ is a vector that represents the activity of all the nodes in the network. *A* is the coupling matrix given by

$$\begin{array}{c} A=(1-\alpha \Delta t)I+C\Delta t, \end{array}$$ (5)

where *I* is the identity matrix, *α* quantifies the leak from each neuron activity (following Ref. [15, 41], we set *α* = 2), and *C* is the coupling matrix that specifies the weighted structural network interaction between the network nodes. In this study, we compare the binarized version of *C* to the thresholded functional networks derived from the network regions’ activation time series.

While these models are simple, it has been shown that such models can have biological significance [46] and produce functional patterns similar to those produced by more complex non-linear models [15].

### Linear diffusion model

This model describes the functional behavior of a network as a diffusion process on the structural network [43]. Specifically, the dynamics of a network with an arbitrary topology is expressed as

$$\begin{array}{c} \frac{dx}{dt}=-\beta Lx\left(t\right)+\eta \left(t\right), \end{array}$$ (6)

where ***x*** is a vector that holds the activity of all nodes, *β* represents the decay rate of the response, *L* is the network Laplacian, and *η*(*t*) represents uncorrelated Gaussian random noise. Following Ref. [43], we define the network Laplacian as

$$\begin{array}{c} L=I-\Delta^{-1/2}C\Delta^{-1/2}, \end{array}$$ (7)

where *I* is the identity matrix, ***C*** is the coupling structural matrix, and Δ is a matrix that has the strength of each node as its diagonal elements.

### Fitzhugh-Nagumo model

The network dynamics can be also simulated by placing Fitzhugh-Nagumo oscillators at each node in the network. In this case, the dynamics of each node is described by two variables: ***u***, which represents the membrane potential of the neurons, and ***v***, which is a recovery variable. We follow the approach outlined in Ref. [35] without implementing the time delay explicitly in the model. As a result, the dynamics of node *i* is given by

$$\begin{array}{c} \begin{array}{cc} & \dot{u_{i}}=\tau (v_{i}+\gamma u_{i}-\frac{u_{i}^{3}}{3})-d\sum_{j=1}^{N}C_{ij}u_{j}+\eta_{u},\\ & v_{i}=-\left(1/\tau \right)\left(u_{i}-\alpha +bv_{i}\right)+\eta_{v}, \end{array} \end{array}$$ (8)

where *C* is the structural matrix between the neurons, *d* is a scaling parameter for the coupling strength, and *η*_*u*_ and *η*_*v*_ represent uncorrelated Gaussian noises that drive the system.

### Definition of the graph measures

The distance *d*_*ij*_ between nodes *i* and *j* in a binary network is the minimum number of edges that need to be traversed in order to reach one node from the other. The path length *L*_*i*_ of node *i* is defined as the average distance from *i* to all other nodes in the network. The characteristic path length *L* of a network is defined as the average of the path lengths of all nodes [37, 76]:

$$\begin{array}{c} L=\frac{1}{N}\sum_{i\in N}L_{i}=\frac{1}{N}\sum_{i\in N}\frac{\sum_{j\in N,j\neq i}d_{ij}}{n-1}. \end{array}$$ (9)

Since the characteristic path length cannot be calculated for disconnected networks, the global efficiency *E*_*i*_ is often employed, which is a related measure that can be meaningfully interpreted on disconnected networks. For a given node, *E*_*i*_ is the average of the inverse distances from that node to all other nodes in the network. The global efficiency of a network *E* is calculated as the average of the global efficiency of all nodes [77]:

$$\begin{array}{c} E=\frac{1}{N}\sum_{i\in N}E_{i}=\frac{1}{N}\sum_{i\in N}\frac{\sum_{j\in N,j\neq i}d^{-}1_{ij}}{n-1}. \end{array}$$ (10)

The clustering coefficient *C*_*i*_ of node *i* reflects the fraction of the neighbors of *i* that are also connected with each other and can be calculated as the fraction of the triangles that are present around *i*. The clustering coefficient of a network *C* is calculated by averaging the clustering coefficients of all nodes: [37, 76]

$$\begin{array}{c} C=\frac{1}{N}\sum_{i\in N}C_{i}=\frac{1}{N}\sum_{i\in N}\frac{2t_{i}}{k_{i}\left(k_{i}-1\right)}, \end{array}$$ (11)

where *t*_*i*_ and *k*_*i*_ are the number of triangles around node *i* and its degree respectively.

The transitivity *T* is a variant of the network’s clustering coefficient that is calculated as the ratio between the number of triangles in the network *τ* and the total number of triplets [78]:

$$\begin{array}{c} T=\frac{3\tau }{\sum_{i\in N}d_{i}\left(d_{i}-1\right)-d_{ii}}, \end{array}$$ (12)

where *d*_*ii*_ represents the false pairs that do not result in triplets. The transitivity is not defined at a nodal level.

Eigenvector centrality is a measure that detects a node’s influence in the network by considering all network paths. A node with high eigenvector centrality will tend to connect with other nodes with high scores. The eigenvector centrality of a node i can be calculated as [79, 80]:

$$\begin{array}{c} EC=\frac{1}{\lambda_{1}}\sum_{j}A_{ij}\nu_{j}, \end{array}$$ (13)

where λ_1_ and ν are the leading eigenvalue and eigenvector of A respectively, and A is the adjacency matrix of the network.

### Data for biological networks

The structural connectomes for the mouse, cat and macaque are obtained from previous studies that used neuronal tracing data to determine the axonal projections between brain regions [49–51]. The structural connectome for the human brain is obtained from a population-averaged atlas of 550,000 white matter trajectories that were clustered and labeled by a team of experienced neuroanatomists in order to conform to prior neuroanatomical knowledge [53].

#### Mouse connectome

The mouse connectome consists of 112 nodes, corresponding to distinct brain regions, with maximum of 53% connection density. The edge weights are defined as the proportion of tracer signal detected in the target region with respect to amount of tracer injected in the source region, which follows a log-normal distribution [49].

#### Cat connectome

The cat connectome has 95 nodes with a maximal density of 23.5%. The weights of the connections are assigned according to their reported density/strengh in the literature; in particular 1 is assigned for weak or sparse connections, 2 for connections with unknown or intermediate strength, and 3 for strong or dense connections [50].

#### Macaque connectome

The macaque connectome consists of a directed subnetwork with 29 nodes and a maximal connection density of 66% that is representative of this animal’s cortico-cortical connections [51, 52]. The weight of the edges was defined by the ratio between the neurons that were labeled by the tracer in the target and source areas relative to the total number of labeled neurons, which followed a log-normal distribution. Our simulations were performed by using an undirected version of this connectome (connection density of 76.5%).

#### Human connectome

The human population-averaged connectome has 65 nodes and a maximal density of 33%. The edge weights were defined as the average of the spin distribution function along the corresponding track [53].

### Ethics statement

The authors of this study did not participate in the data acquisition. All human and animal data used in the current study was made publicly available by the corresponding studies [49–53]. Ethical approval to collect human and animal data was received at the research centers where the data was acquired.

Ref. [53] used a minimally pre-processed human data from the Human Connectome Project (Q1-Q4 release, 2015) acquired by Washington University in Saint Louis and University of Minnesota [81]. The macaque data was obtained by ref. [51, 52] in accordance with European requirements 86/609/EEC and approved by the ethics committee of the region Rhône-Alpes. Ref. [49] used mouse data that was made available as part of the Allen Institute Mouse Brain Connectivity Atlas where all experiments were approved by the Institutional Animal Care and Use Committee of the Allen Institute for Brain Science, in accordance with NIH guidelines [48]. The cat connectivity was calculated in University of Newcastle and Oxford University by collating publicly available data on the cortico-thalamic system of the cat [50].

## Supporting information

S1 Appendix This appendix contains tables A-P and figs A-C. (PDF)

S1 Data Set of scripts and structural matrices used in the analysis. (ZIP)
