# Supplementary material for: Delayed correlations improve the reconstruction of the brain connectome
Source: PLoS One. 2020 Feb 19;15(2):e0228334. doi: 10.1371/journal.pone.0228334 (PMC7029855; doi:10.1371/journal.pone.0228334)
Supplement: S1 Appendix — (PDF) [file pone.0228334.s001.pdf]

Delayed correlations improve the reconstruction of the brain connectome

Supplementary information

Mite Mijalkov and Joana B. Pereira
Department of Neurobiology, Care Sciences and Society, Karolinska Institutet, Stockholm, Sweden

Giovanni Volpe
Department of Physics, University of Gothenburg, Gothenburg, Sweden

I. IDENTIFIED DIRECT AND INDIRECT CONNECTIONS FOR DIFFERENT NETWORK SIZES AND DENSITIES

TABLE A. Accuracy of network reconstruction. Percentage of correctly identified edges in structural networks for different network sizes (100, 200 and 500 nodes) and densities (2% to 14%). The means are followed by standard deviations calculated over 100 trials. The structural networks are small-world networks with $\beta = 0.05$, and the connection strengths are drawn from a symmetric q-Gaussian distribution with $q = 1$, as in Fig. 2.

			**100 nodes network**				
Density	2%	4%	6%	8%	10%	12%	14%
delayed correlation	81.3(4.3)	76.1(3.7)	72.1(3.5)	68.8(3.2)	66.5(2.6)	63.9(2.7)	62.4(2.4)
absolute correlation	1.8(1.4)	14.3(2.9)	18.1(2.9)	19.9(2.7)	21.8(2.1)	22.8(2.2)	24.4(2.1)
same-time correlation	1.9(1.3)	10.2(2.3)	13.4(2.0)	15.1(1.9)	16.8(1.6)	18.2(1.8)	19.8(1.5)
null model	2.1(1.5)	4.2(1.4)	5.9(1.2)	8.2(1.3)	10.3(1.3)	12.1(1.2)	14.2(1.3)
			**200 nodes network**				
Density	2%	4%	6%	8%	10%	12%	14%
delayed correlation	75.1(2.7)	67.1(2.9)	61.6(2.3)	57.6(2.2)	53.9(2.1)	51.8(1.5)	49.7(1.4)
absolute correlation	9.6(2.2)	13.3(2.2)	15.0(1.5)	16.4(1.5)	17.8(1.2)	19.3(0.9)	20.6(0.9)
same-time correlation	6.9(1.8)	9.8(1.5)	11.6(1.1)	13.1(1.1)	14.7(0.9)	16.3(0.8)	17.7(0.6)
null model	2.0(0.8)	3.9(0.6)	6.1(0.7)	8.1(0.6)	10.0(0.7)	12.2(0.6)	14.1(0.5)
			**500 nodes network**				
Density	2%	4%	6%	8%	10%	12%	14%
delayed correlation	62.5(2.3)	51.5(1.4)	45.2(1.1)	39.9(1.2)	36.7(0.9)	34.5(0.7)	33.2(0.7)
absolute correlation	8.2(1.2)	9.4(0.6)	11.2(0.6)	12.4(0.6)	13.8(0.5)	15.5(0.4)	17.2(0.3)
same-time correlation	6.2(0.9)	7.6(0.5)	9.4(0.5)	10.9(0.4)	12.4(0.3)	14.1(0.3)	15.9(0.3)
null model	2.0(0.3)	3.9(0.2)	6.0(0.2)	7.9(0.3)	10.0(0.2)	12.0(0.2)	13.9(0.2)

TABLE B. Percentage of 2-, 3- and 4-edge indirect connections incorrectly identified as direct connections for different network sizes (100, 200 and 500 nodes) and densities (2% to 14%). The correctly identified direct connections are also included for comparison. The means are followed by standard deviations calculated over 100 trials. The structural networks are small-world networks with $\beta = 0.05$, and the connection strengths are drawn from a symmetric q-Gaussian distribution with $q = 1$, as in Fig. 2.

**100 nodes network**			**Delayed correlation**				
Density	2%	4%	6%	8%	10%	12%	14%
direct connections	81.3(4.3)	76.1(3.7)	72.1(3.5)	68.8(3.2)	66.5(2.6)	63.9(2.7)	62.4(2.4)
2-edge indirect connections	9.6(3.3)	8.9(2.7)	7.4(1.9)	7.3(1.4)	8.6(1.5)	10.7(1.5)	13.2(1.5)
3-edge indirect connections	0.3(0.6)	1.3(0.8)	3.7(1.5)	8.1(1.9)	12.9(2.4)	18.3(2.2)	20.9(2.1)
4-edge indirect connections	0.2(0.5)	1.6(0.9)	5.2(2.0)	8.9(2.0)	9.6(1.7)	6.8(1.9)	3.5(1.7)
>4-edge indirect connections	8.6(3.8)	12.0(4.4)	11.6(3.5)	6.8(2.6)	2.3(1.7)	0.3(0.6)	0(0.1)
			**Absolute correlation**				
Density	2%	4%	6%	8%	10%	12%	14%
direct connections	1.8(1.4)	14.3(2.9)	18.1(2.9)	19.9(2.7)	21.8(2.1)	22.8(2.2)	24.4(2.1)
2-edge indirect connections	20.9(5.9)	18.8(3.9)	19.5(3.2)	21.1(2.8)	24.4(2.9)	28.1(2.6)	31.3(2.5)
3-edge indirect connections	1.7(1.2)	5.5(1.8)	11.4(2.5)	19.9(3.6)	27.9(3.7)	35.6(3.4)	37.9(2.9)
4-edge indirect connections	1.9(1.5)	8.1(2.3)	15.5(3.5)	22.1(3.3)	20.8(2.7)	12.8(3.3)	6.3(2.8)
>4-edge indirect connections	73.7(6.0)	53.4(5.7)	35.6(6.3)	16.9(5.9)	5.0(3.2)	0.7(1.3)	0.0(0.2)
			**Same-time correlation**				
Density	2%	4%	6%	8%	10%	12%	14%
direct connections	1.9(1.3)	10.2(2.3)	13.4(2.0)	15.1(1.9)	16.8(1.6)	18.2(1.8)	19.8(1.5)
2-edge indirect connections	12.6(4.1)	13.7(2.9)	15.5(2.3)	18.2(2.6)	22.2(2.6)	26.3(2.6)	29.9(2.4)
3-edge indirect connections	1.9(1.4)	6.4(1.9)	12.7(2.6)	22.6(3.9)	31.7(4.2)	39.9(3.2)	42.9(2.7)
4-edge indirect connections	2.1(1.5)	9.1(2.7)	17.7(4.2)	25.2(3.3)	23.7(2.7)	14.8(3.5)	7.2(3.1)
>4-edge indirect connections	81.5(4.2)	60.7(4.8)	40.7(6.9)	18.9(6.2)	5.6(3.7)	0.8(1.3)	0.0(0.1)
**200 nodes network**			**Delayed correlation**				
Density	2%	4%	6%	8%	10%	12%	14%
direct connections	75.1(0.8)	67.1(0.6)	61.6(0.7)	57.6(0.6)	53.9(0.7)	51.8(0.6)	49.7(0.5)
2-edge indirect connections	6.6(2.1)	4.9(0.9)	6.3(0.8)	9.6(0.9)	13.4(1.1)	18.2(1.1)	23.5(1.4)
3-edge indirect connections	0.7(0.4)	5.1(1.1)	14.4(1.7)	24.1(1.9)	29.8(1.8)	29.6(1.3)	26.8(1.3)
4-edge indirect connections	1.2(0.4)	8.2(1.1)	13.8(1.7)	8.5(1.9)	2.8(1.8)	0.4(1.3)	0.1(1.3)
>4-edge indirect connections	16.5(4.0)	14.6(2.3)	3.9(1.6)	0.2(0.2)	0(0)	0(0)	0(0)
			**Absolute correlation**				
Density	2%	4%	6%	8%	10%	12%	14%
direct connections	9.6(2.2)	13.3(2.2)	15.0(1.5)	16.4(1.5)	17.8(1.2)	19.3(0.9)	20.6(0.9)
2-edge indirect connections	12.3(2.7)	13.8(1.8)	17.7(1.9)	22.1(1.6)	27.4(1.7)	33.6(1.6)	39.5(1.6)
3-edge indirect connections	3.1(0.9)	13.2(1.8)	29.8(2.9)	45.2(2.8)	50.1(1.9)	46.5(1.6)	39.9(1.6)
4-edge indirect connections	4.8(1.3)	21.5(3.4)	29.3(2.1)	15.8(2.9)	4.7(1.9)	0.6(0.4)	0.1(0.1)
>4-edge indirect connections	70.1(4.5)	38.2(4.3)	8.2(3.3)	0.4(0.4)	0(0.1)	0(0)	0(0)
			**Same-time correlation**				
Density	2%	4%	6%	8%	10%	12%	14%
direct connections	6.9(1.8)	9.8(1.5)	11.6(1.1)	13.1(1.1)	14.7(0.9)	16.3(0.8)	17.7(0.6)
2-edge indirect connections	9.1(1.8)	11.5(1.5)	15.9(1.5)	21.2(1.5)	26.9(1.8)	33.2(1.7)	39.8(1.7)
3-edge indirect connections	3.4(0.9)	14.6(1.9)	32.3(3.0)	48.3(2.8)	53.4(1.9)	49.8(1.6)	42.5(1.6)
4-edge indirect connections	5.2(1.4)	23.1(3.3)	31.4(2.2)	16.9(3.2)	5.0(1.9)	0.6(0.5)	0.1(0.1)
>4-edge indirect connections	75.4(3.2)	41.1(4.7)	8.7(3.5)	0.4(0.4)	0(0.1)	0(0)	0(0)
**500 nodes network**			**Delayed correlation**				
Density	2%	4%	6%	8%	10%	12%	14%
direct connections	62.5(2.3)	51.5(1.4)	45.2(1.1)	39.9(1.2)	36.7(0.9)	34.5(0.7)	33.2(0.7)
2-edge indirect connections	3.1(0.4)	6.3(0.4)	12.6(0.5)	21.2(0.9)	30.6(0.9)	40.2(1.1)	48.4(0.9)
3-edge indirect connections	4.4(0.6)	25.9(1.3)	40.4(0.9)	38.8(1.1)	32.7(1.1)	25.3(0.9)	18.3(0.8)
4-edge indirect connections	8.9(1.1)	15.7(1.2)	1.9(0.5)	0.1(0.1)	0(0)	0(0)	0(0)
>4-edge indirect connections	21.1(2.0)	0.6(0.3)	0(0)	0(0)	0(0)	0(0)	0(0)
			**Absolute correlation**				
Density	2%	4%	6%	8%	10%	12%	14%
direct connections	8.2(1.2)	9.4(0.6)	11.2(0.6)	12.4(0.6)	13.8(0.5)	15.5(0.4)	17.2(0.3)
2-edge indirect connections	7.9(0.8)	13.8(0.7)	22.5(0.8)	32.6(1.2)	42.8(1.1)	52.7(1.1)	60.6(0.9)
3-edge indirect connections	10.9(1.1)	47.3(1.9)	63.3(0.9)	54.9(1.3)	43.4(1.1)	31.8(1.1)	22.2(0.9)
4-edge indirect connections	21.9(2.1)	28.4(1.9)	2.9(0.7)	0.1(0.1)	0(0)	0(0)	0(0)
>4-edge indirect connections	51.2(3.0)	1.1(0.5)	0(0)	0(0)	0(0)	0(0)	0(0)
			**Same-time correlation**				
Density	2%	4%	6%	8%	10%	12%	14%
direct connections	6.2(0.9)	7.6(0.5)	9.4(0.5)	10.9(0.4)	12.4(0.3)	14.1(0.3)	15.9(0.3)
2-edge indirect connections	6.7(0.7)	13.1(0.7)	22.1(0.8)	32.5(1.2)	43.0(1.2)	53.2(1.1)	61.3(0.9)
3-edge indirect connections	11.0(1.0)	48.8(1.9)	65.5(0.9)	56.6(1.3)	44.6(1.2)	32.7(1.1)	22.8(0.9)
4-edge indirect connections	22.8(2.1)	29.3(1.9)	3.1(0.7)	0.1(0.1)	0(0)	0(0)	0(0)
>4-edge indirect connections	53.3(3.1)	1.2(0.5)	0(0)	0(0)	0(0)	0(0)	0(0)

II. NETWORK RECONSTRUCTION WITH DIFFERENT NODAL ACTIVATION DYNAMICS

A. Linear Diffusion model

TABLE C. Accuracy of network reconstruction when the dynamics of each network node is simulated by the Linear Diffusion model. Percentage of correctly identified edges in 200-node networks with density from 2% to 14%. The means are followed by standard deviations and calculated over 100 trials. The structural networks are small-world networks with $\beta = 0.05$, and the connection strengths are drawn from a symmetric q-Gaussian distribution with $q = 1$.

Density	2%	4%	6%	8%	10%	12%	14%
delayed correlation	73.1(2.2)	90.0(1.0)	89.8(0.8)	79.0(1.1)	65.7(1.3)	53.9(1.0)	44.4(1.0)
absolute correlation	47.5(2.5)	57.4(2.2)	49.3(2.0)	41.8(1.7)	36.9(1.6)	33.8(1.2)	31.7(1.0)
same-time correlation	47.6(2.4)	59.2(2.2)	53.1(2.0)	46.7(1.8)	42.3(1.6)	39.8(1.2)	37.7(1.1)
null model	1.9(0.7)	4.1(0.8)	6.1(0.7)	8.2(0.7)	10.1(0.6)	11.9(0.6)	14.1(0.6)

B. Fitzhugh-Nagumo model

TABLE D. Accuracy of network reconstruction when the dynamics of each network node is simulated by the Fitzhugh-Nagumo model. Percentage of correctly identified edges in 200-node networks with density from 2% to 14%. The means are followed by standard deviations and calculated over 100 trials. The structural networks are small-world networks with $\beta = 0.05$, and the connection strengths are drawn from a symmetric q-Gaussian distribution with $q = 1$.

Density	2%	4%	6%	8%	10%	12%	14%
delayed correlation	66.2(6.7)	60.7(8.3)	57.8(5.5)	55.2(5.1)	51.7(9.0)	50.9(6.0)	49.5(4.1)
absolute correlation	63.8(6.6)	58.7(8.2)	56.4(5.8)	54.2(5.3)	50.9(9.2)	50.5(6.5)	49.9(4.8)
same-time correlation	39.0(4.4)	36.5(5.0)	35.6(3.6)	34.4(3.2)	32.9(5.3)	33.2(3.7)	33.1(2.7)
null model	2.1(0.7)	4.0(0.8)	6.0(0.7)	8.0(0.7)	10.1(0.7)	12.1(0.7)	14.2(0.6)

III. GLOBAL MEASURES

TABLE E. Global network measures in reconstructed networks as a function of network density. Characteristic path length, global efficiency, clustering coefficient and transitivity of the underlying structural networks with 200 nodes and density in the range from 2% to 14% and the corresponding reconstructed networks using the four methods. The means are followed by standard deviations calculated over 100 trials. The structural networks are small-world networks with $\beta = 0.05$, and the connection strengths are drawn from a symmetric q-Gaussian distribution with $q = 1$. The network activation dynamics was simulated with linearized Wilson-Cowan model. Compare with Fig. 3.

			**Characteristic path length**				
Density	2%	4%	6%	8%	10%	12%	14%
structural network	8.13(0.85)	4.29(0.18)	3.31(0.09)	2.85(0.05)	2.60(0.03)	2.44(0.02)	2.32(0.02)
delayed correlation	4.83(0.26)	2.96(0.04)	2.49(0.01)	2.24(0.01)	2.06(0.01)	1.95(0)	1.88(0)
absolute correlation	3.92(0.05)	2.77(0.01)	2.40(0)	2.18(0)	2.02(0)	1.92(0)	1.86(0)
same-time correlation	3.93(0.06)	2.77(0.01)	2.40(0)	2.18(0)	2.02(0)	1.93(0)	1.88(0)
null model	3.92(0.04)	2.77(0.01)	2.40(0)	2.17(0)	2.02(0)	1.93(0)	1.86(0)
			**Global efficiency**				
Density	2%	4%	6%	8%	10%	12%	14%
structural network	0.17(0)	0.28(0)	0.35(0)	0.40(0)	0.44(0)	0.47(0)	0.49(0)
delayed correlation	0.24(0.01)	0.37(0)	0.44(0)	0.49(0)	0.52(0)	0.55(0)	0.57(0)
absolute correlation	0.27(0)	0.39(0)	0.45(0)	0.50(0)	0.53(0)	0.55(0)	0.57(0)
same-time correlation	0.28(0)	0.39(0)	0.45(0)	0.50(0)	0.53(0)	0.55(0)	0.57(0)
null model	0.28(0)	0.39(0)	0.45(0)	0.50(0)	0.53(0)	0.55(0)	0.57(0)
			**Clustering coefficient**				
Density	2%	4%	6%	8%	10%	12%	14%
structural network	0.44(0)	0.56(0)	0.59(0)	0.61(0)	0.62(0)	0.62(0)	0.63(0)
delayed correlation	0.27(0.04)	0.22(0.03)	0.18(0.02)	0.17(0.01)	0.17(0.01)	0.17(0.01)	0.18(0)
absolute correlation	0.03(0.01)	0.05(0.01)	0.07(0)	0.08(0)	0.10(0)	0.12(0)	0.14(0)
same-time correlation	0.03(0.01)	0.05(0.01)	0.07(0)	0.09(0)	0.11(0)	0.13(0)	0.15(0)
null model	0.02(0.01)	0.04(0)	0.06(0)	0.08(0)	0.10(0)	0.12(0)	0.14(0)
			**Transitivity**				
Density	2%	4%	6%	8%	10%	12%	14%
structural network	0.43(0)	0.55(0)	0.58(0)	0.60(0)	0.61(0)	0.62(0)	0.63(0)
delayed correlation	0.25(0.04)	0.20(0.03)	0.17(0.02)	0.17(0.01)	0.16(0)	0.17(0)	0.18(0)
absolute correlation	0.03(0.01)	0.05(0.01)	0.07(0)	0.08(0)	0.10(0)	0.12(0)	0.14(0)
same-time correlation	0.03(0.01)	0.05(0)	0.07(0)	0.09(0)	0.11(0)	0.13(0)	0.15(0)
null model	0.02(0.01)	0.04(0)	0.06(0)	0.08(0)	0.10(0)	0.12(0)	0.14(0)

IV. NODAL MEASURES

TABLE F. Nodal network measures in reconstructed networks as a function of network density. Average percentage of correctly reconstructed nodal degree, clustering coefficient and global efficiency using the four methods for networks with 200 nodes and density from 2% to 14%. The means are followed by standard deviations calculated over 100 trials. The structural networks are small-world networks with $\beta = 0.05$, and the connection strengths are drawn from a symmetric q-Gaussian distribution with $q = 1$. Compare with Fig. 4.

			**Degree**				
Density	2%	4%	6%	8%	10%	12%	14%
delayed correlation	73.6(2.0)	78.5(1.4)	81.5(1.1)	83.7(1.0)	84.9(0.8)	86.0(0.7)	87.0(0.7)
absolute correlation	59.5(2.2)	71.8(1.6)	76.8(1.3)	80.0(1.2)	82.4(0.9)	84.1(0.8)	85.5(0.8)
same-time correlation	60.1(2.2)	71.8(1.6)	77.2(1.2)	80.4(1.1)	82.7(1.1)	84.3(0.9)	85.6(0.8)
null model	60.2(2.2)	72.2(1.7)	77.3(1.1)	80.5(1.2)	82.8(1.0)	84.4(0.9)	85.7(0.7)
			**Global efficiency**				
Density	2%	4%	6%	8%	10%	12%	14%
delayed correlation	51.6(10.5)	65.9(3.6)	74.5(2.5)	78.8(1.5)	80.3(1.1)	82.5(0.7)	85.1(0.6)
absolute correlation	26.3(11.3)	58.1(4.1)	70.5(2.5)	75.9(1.6)	78.8(1.1)	81.7(0.7)	84.8(0.6)
same-time correlation	26.3(11.3)	58.2(4.2)	70.5(2.5)	76.0(1.6)	78.9(1.1)	81.8(0.7)	84.8(0.6)
null model	25.6(11.4)	57.9(4.2)	70.4(2.4)	75.8(1.6)	78.7(1.1)	81.7(0.7)	84.8(0.6)
			**Clustering coefficient**				
Density	2%	4%	6%	8%	10%	12%	14%
delayed correlation	41.6(5.8)	38.8(4.9)	31.4(3.0)	28.4(2.2)	27.1(1.6)	27.5(1.0)	28.4(0.7)
absolute correlation	5.0(1.5)	8.6(1.0)	11.3(0.8)	14.1(0.5)	16.9(0.5)	19.9(0.4)	22.8(0.4)
same-time correlation	4.8(1.5)	8.9(1.1)	11.9(0.8)	15.1(0.5)	18.0(0.6)	21.1(0.5)	24.1(0.4)
null model	3.5(1.4)	7.3(0.9)	10.5(0.6)	13.4(0.6)	16.4(0.6)	19.5(0.4)	22.6(0.4)
			**Eigenvector centrality**				
Density	2%	4%	6%	8%	10%	12%	14%
delayed correlation	37.7(8.1)	68.2(3.7)	76.2(2.4)	80.8(1.8)	83.2(1.4)	84.8(1.4)	86.3(1.1)
absolute correlation	37.4(9.6)	64.8(3.4)	73.0(2.3)	78.0(1.9)	81.1(1.5)	83.1(1.3)	85.0(1.1)
same-time correlation	38.5(8.8)	64.2(3.2)	73.2(2.3)	77.8(1.8)	81.0(1.4)	83.2(1.4)	84.9(1.1)
null model	37.5(10.3)	64.9(2.9)	73.7(2.2)	78.2(1.8)	81.4(1.4)	83.5(1.4)	85.1(1.1)
			**PageRank centrality**				
Density	2%	4%	6%	8%	10%	12%	14%
delayed correlation	78.5(1.5)	81.9(1.1)	84.4(0.9)	86.2(0.8)	87.2(0.7)	88.1(0.6)	88.9(0.6)
absolute correlation	66.7(1.7)	76.3(1.3)	80.4(1.0)	83.0(1.0)	85.1(0.7)	86.5(0.7)	87.7(0.7)
same-time correlation	67.1(1.8)	76.3(1.3)	80.8(1.0)	83.4(1.0)	85.3(0.9)	86.7(0.8)	87.8(0.6)
null model	67.0(1.7)	76.6(1.4)	80.8(0.9)	83.5(1.0)	85.4(0.8)	86.8(0.7)	87.8(0.5)
			**Laplacian centrality**				
Density	2%	4%	6%	8%	10%	12%	14%
delayed correlation	62.5(2.7)	71.1(1.8)	75.3(1.5)	78.3(1.3)	80.0(1.1)	81.5(1.0)	82.8(0.9)
absolute correlation	46.9(2.9)	63.1(2.1)	69.6(1.7)	73.7(1.5)	76.8(1.2)	79.0(1.1)	80.9(1.1)
same-time correlation	47.9(3.0)	63.0(2.0)	69.9(1.6)	74.2(1.4)	77.0(1.3)	79.2(1.2)	80.9(1.0)
null model	48.0(2.7)	63.6(2.0)	70.3(1.5)	74.4(1.5)	77.4(1.3)	79.5(1.1)	81.1(0.9)

V. RECONSTRUCTION OF THE DEGREE DISTRIBUTION

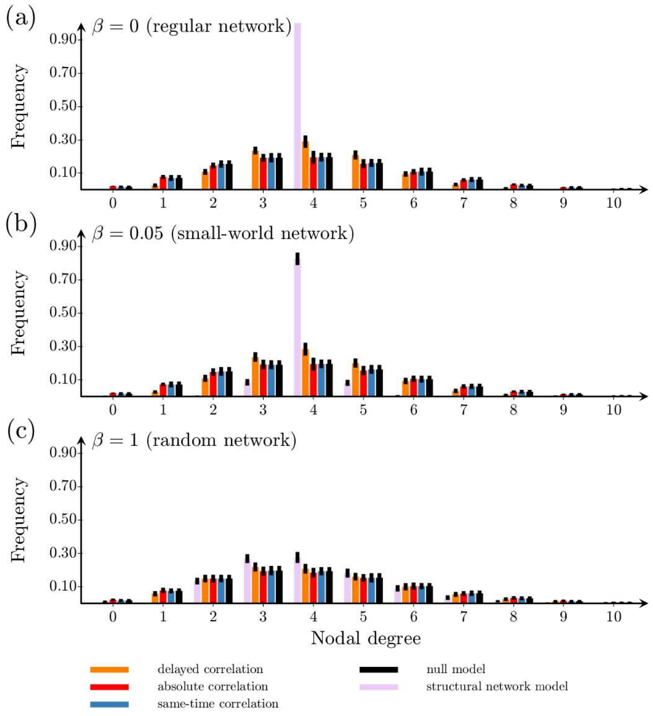

FIG. A. **Degree distribution.** Degree distribution of the networks reconstructed by the delayed-correlation method (orange bars), the absolute correlation method (red bars) and the same time correlation method (blue bars) for a (a) regular network, (b) small-world network and (c) random network. The violet bars show the degree distribution for the structural network while the black bars represent the null model. Each bar denotes the average of 100 simulations; the error bars represent one standard deviation. All networks have 200 nodes and were thresholded at 2% density. The connection strengths are drawn from a symmetric q-Gaussian distribution with $q = 1$ and the network dynamics were simulated by the linear Wilson-Cowan model.

VI. ROBUSTNESS OF THE DELAYED-CORRELATION METHOD FOR ALTERNATIVE DYNAMICS MODELS

A. Linear Diffusion model

TABLE G. Reconstruction performance for various kinds of networks with dynamics simulated according to a Linear Diffusion model. Reconstruction efficiency as a function of the probability to randomize an edge in the Watts-Strogatz model. The means are followed by standard deviations and calculated over 100 trials. The structural networks have 200 nodes and 2% density, and the connection strengths are drawn from a symmetric q-Gaussian distribution with $q = 1$.

Beta	0	0.10	0.20	0.30	0.40	0.50
delayed correlation	74.7(2.4)	71.3(2.5)	68.8(2.6)	66.8(2.9)	65.7(3.1)	64.7(3.1)
absolute correlation	52.9(1.6)	40.9(2.9)	28.8(3.2)	19.2(2.8)	11.8(2.3)	7.2(2.0)
same-time correlation	52.9(1.7)	41.0(2.9)	29.0(3.2)	19.5(2.8)	12.0(2.3)	7.3(2.1)
null model	2.0(0.7)	2.1(0.7)	2.1(0.8)	2.0(0.7)	2.0(0.7)	1.9(0.7)
Beta	0.60	0.70	0.80	0.90	1	
delayed correlation	63.9(3.0)	64.3(2.9)	64.3(2.9)	64.2(2.6)	64.2(2.7)	
absolute correlation	4.0(1.4)	2.7(1.1)	1.7(0.8)	1.4(0.7)	1.5(0.7)	
same-time correlation	4.1(1.4)	2.7(1.1)	1.7(0.8)	1.3(0.7)	1.4(0.8)	
null model	2.1(0.8)	2.0(0.7)	2.0(0.7)	2.2(0.6)	2.1(0.7)	

TABLE H. Reconstruction performance for various kinds of networks with dynamics simulated according to a Linear Diffusion model. Reconstruction efficiency as a function of the different distribution of weights in the structural network represented by the q parameter in a q-Gaussian distribution. The means are followed by standard deviations and calculated over 100 trials. The structural networks are small-world networks with $\beta = 0.05$, have 200 nodes and 2% density.

q	- 3	- 2	- 1	0	1	2	3
delayed correlation	70.3(2.4)	69.9(2.3)	69.7(2.6)	69.4(2.4)	73.2(2.4)	76.7(2.7)	75.1(2.6)
absolute correlation	49.3(2.1)	49.4(1.9)	49.4(1.9)	49.7(2.0)	47.2(2.5)	45.2(2.8)	46.0(2.7)
same-time correlation	49.3(2.1)	49.5(2.0)	49.5(1.9)	49.8(2.0)	47.2(2.5)	45.5(2.9)	46.2(2.7)
null model	2.0(0.7)	2.0(0.7)	2.1(0.7)	2.1(0.7)	2.1(0.7)	2.0(0.7)	2.0(0.7)

B. Fitzhugh-Nagumo model

TABLE I. Reconstruction performance for various kinds of networks with dynamics simulated according to the Fitzhugh-Nagumo model. Reconstruction efficiency as a function of the probability to randomize an edge in the Watts-Strogatz model. The means are followed by standard deviations and calculated over 100 trials. The structural networks have 200 nodes and 2% density, and the connection strengths are drawn from a symmetric q-Gaussian distribution with $q = 1$.

Beta	0	0.10	0.20	0.30	0.40	0.50
delayed correlation	67.5(6.8)	66.4(1.8)	61.9(13.9)	62.5(8.9)	60.9(12.2)	62.5(6.3)
absolute correlation	66.2(6.7)	62.5(2.5)	56.7(12.7)	56.2(8.1)	53.9(10.8)	54.3(5.7)
same-time correlation	39.7(4.5)	38.6(2.2)	36.3(8.2)	35.7(5.3)	34.2(6.9)	34.7(3.7)
null model	2.0(0.7)	2.1(0.7)	2.1(0.7)	1.9(0.7)	2.1(0.7)	2.0(0.7)

Beta	0.60	0.70	0.80	0.90	1
delayed correlation	60.3(10.4)	59.9(10.5)	59.4(11.9)	58.9(13.3)	61.3(6.3)
absolute correlation	52.6(9.2)	52.9(9.3)	51.9(10.4)	50.9(11.4)	53.4(5.6)
same-time correlation	33.1(6.1)	33.3(5.9)	32.9(6.7)	32.7(7.5)	34.0(3.9)
null model	2.1(0.7)	1.9(0.7)	2.1(0.7)	1.9(0.7)	2.0(0.6)

TABLE J. Reconstruction performance for various kinds of networks with dynamics simulated according to the Fitzhugh-Nagumo model. Reconstruction efficiency as a function of the percentage of positive weights in the weighted structural network. The means are followed by standard deviations and calculated over 100 trials. The structural networks are small-world networks with $\beta = 0.05$, have 200 nodes and 2% density, and the connection strengths are drawn from a symmetric q-Gaussian distribution with $q = 1$.

Percentages	0 %	10 %	20 %	30 %	40 %	50 %
delayed correlation	66.3(13.4)	61.9(8.7)	58.2(5.9)	57.1(5.9)	59.6(6.1)	65.2(2.5)
absolute correlation	64.9(13.0)	62.8(8.9)	60.7(6.2)	60.1(6.2)	61.6(6.3)	63.8(2.2)
same-time correlation	64.9(13.0)	62.4(8.9)	58.0(5.9)	50.2(5.2)	43.7(4.6)	39.1(1.6)
null model	2.0(0.8)	1.9(0.6)	1.9(0.6)	2.0(0.7)	1.9(0.7)	1.9(0.7)

Percentages	60 %	70 %	80 %	90 %	100 %
delayed correlation	59.5(2.3)	55.4(5.7)	54.9(1.8)	54.3(7.7)	59.8(2.5)
absolute correlation	62.2(2.1)	59.5(6.1)	59.4(1.9)	58.9(8.3)	60.8(1.9)
same-time correlation	30.4(1.7)	22.1(2.6)	16.3(1.8)	11.6(2.1)	8.5(1.8)
null model	2.0(0.7)	1.9(0.6)	2.1(0.7)	1.9(0.9)	1.9(0.6)

TABLE K. Reconstruction performance for various kinds of networks with dynamics simulated according to the Fitzhugh-Nagumo model. Reconstruction efficiency as a function of the different distribution of weights in the structural network represented by by the q parameter in a q-Gaussian distribution. The means are followed by standard deviations and calculated over 100 trials. The structural networks are small-world networks with $\beta = 0.05$, have 200 nodes and 2% density.

q	- 3	- 2	- 1	0	1	2	3
delayed correlation	66.3(6.7)	64.0(11.0)	64.7(6.5)	61.8(8.7)	66.5(6.8)	58.1(10.3)	62.7(12.5)
absolute correlation	61.4(6.3)	59.8(10.4)	60.7(6.2)	58.8(8.3)	63.8(6.5)	60.2(10.4)	60.8(12.2)
same-time correlation	38.5(4.3)	37.7(6.6)	38.6(4.4)	36.4(5.4)	39.0(4.3)	35.1(6.3)	37.3(7.6)
null model	2.1(0.6)	2.1(0.7)	2.0(0.7)	1.9(0.7)	2.1(0.7)	2.0(0.7)	2.1(0.7)

VII. RECONSTRUCTION OF NETWORK COMMUNITY STRUCTURE

The community structure and modularity are important properties that characterize the organization of a network into communities or modules [1, 2]. In order to assess the performance of the various reconstruction methods in predicting these properties, we build a highly modular network of 200 nodes joining five unconnected 40-node communities (modularity $m = 0.8$), and then we decrease its modularity by rewiring up to 25% of its connections (Fig. Ba), which results in an average network modularity (over 100 networks) of $m = 0.61$. For the entire modularity range, the delayed correlation method performs better than standard methods, successfully predicting around 65% of connections in the structural network (Fig. Bb). To quantify the quality of the reconstruction of the community structure, we calculate the percentage of node pairs that are in the same community as in the underlying structural network (Fig. Bc): For highly modular networks, the delayed correlation method correctly predicts the community assignment for up to 90% node pairs, while the other methods can predict only up to 20%. For lower modular networks, for example with 25% random connections, the delayed correlation method predicts 50% of the community structure in average, but with large variations. These variations in the performance of the delayed correlation method support the findings that the community structure of the structural network plays an important role in shaping the community structure of the functional networks [3, 4], and, in particular, suggest that highly modular networks tend to produce functional patterns that more closely reflect their community structure.

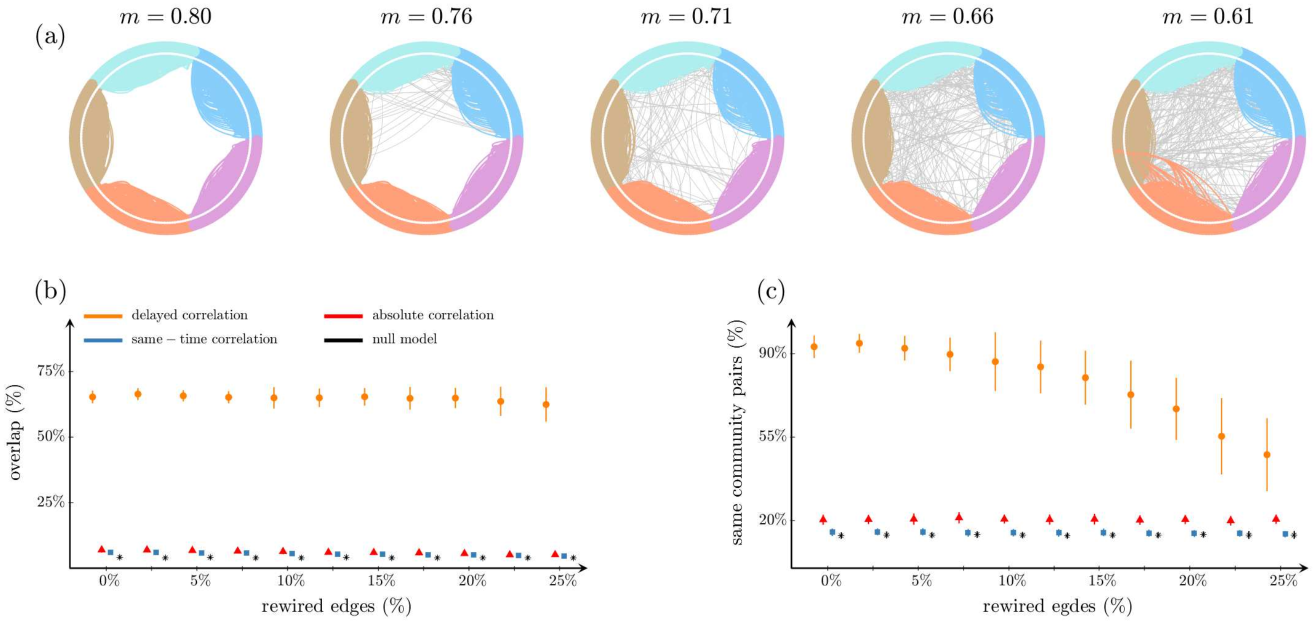

FIG. B. **Reconstruction of the community structure.** (a) A modular network of 200 nodes (4% density) is built by joining 5 distinct, equal-sized networks. When no inter-modular connections are added, the modularity reaches its maximum value of $m = 0.8$ (leftmost network). As randomly selected connections are rewired in the network (gray connections), the modularity decreases. (b) Percentage of overlap (i.e., correctly reconstructed connections) between the structural network and the reconstructed network and (c) percentage of node pairs that belong to the same community in the structural network and in the reconstructed network using the delayed correlation method (orange), the absolute correlation method (red), the same-time correlation method (blue), and the null model (black) as a function of the percentage of randomly rewired connections.

VIII. ROBUSTNESS OF THE DELAYED-CORRELATION METHOD: DEPENDENCE ON NOISE INTENSITY

A. Wilson-Cowan model

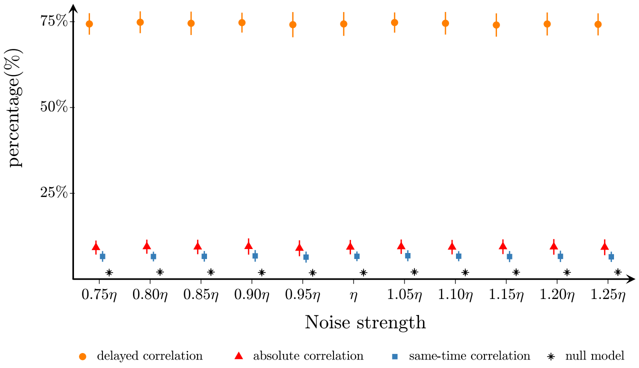

FIG. C. **Performance of reconstruction methods for different intensities of random noise.** The reconstruction efficiency is shown as a function of noise intensity in the Wilson-Cowan model. The results are averaged over 100 trials and the error bars represent one standard deviation. All networks have 200 nodes and were thresholded at 2% density. The connection strengths are drawn from a symmetric q-Gaussian distribution with $q = 1$. η represents the strength of the noise used to obtain the results presented in the main text.

B. Linear Diffusion model

TABLE L. Reconstruction efficiency as a function of noise intensity in the diffusion model. The means are followed by standard deviations and calculated over 100 trials. All networks have 200 nodes and were thresholded at 2% density. The connection strengths are drawn from a symmetric q-Gaussian distribution with $q = 1$. η represents the strength of the noise used to obtain the results presented in the main text.

Noise strength	0.75η	0.80η	0.85η	0.90η	0.95η	η	1.05η
delayed correlation	73.2(2.3)	73.6(2.2)	73.3(2.5)	73.1(2.3)	73.3(2.5)	73.4(2.2)	73.3(2.7)
absolute correlation	47(2.4)	47.3(2.1)	47.5(2.7)	47.3(2.4)	46.9(2.6)	47.4(2.3)	47.5(2.5)
same-time correlation	47(2.4)	47.4(2.1)	47.5(2.7)	47.4(2.4)	47(2.6)	47.5(2.3)	47.5(2.5)
null model	1.9(0.6)	2.1(0.7)	2(0.7)	1.9(0.7)	1.8(0.7)	1.9(0.6)	2.1(0.7)

Noise strength	1.10η	1.15η	1.20η	1.25η
delayed correlation	72.9(2.6)	73.1(2.4)	72.7(2.4)	73.5(2.1)
absolute correlation	47.5(2.6)	47(2.5)	46.8(2.6)	47.2(2.4)
same-time correlation	47.6(2.6)	47.1(2.5)	46.9(2.5)	47.2(2.4)
null model	1.9(0.7)	2(0.7)	2(0.6)	2(0.7)

C. FitzHugh-Nagumo model

TABLE M. Reconstruction efficiency as a function of noise intensity in the FitzHugh-Nagumo model. The means are followed by standard deviations and calculated over 100 trials. All networks have 200 nodes and were thresholded at 2% density. The connection strengths are drawn from a symmetric q-Gaussian distribution with $q = 1$. η represents the strength of the noise used to obtain the results presented in the main text.

Noise strength	0.75η	0.80η	0.85η	0.90η	0.95η	η	1.05η
delayed correlation	64.2(1.9)	65.3(1.7)	65.5(1.8)	65.4(1.8)	66.5(2.3)	66.6(2)	66.5(2.2)
absolute correlation	61.9(2.2)	62.4(2)	63.3(2)	63.6(2)	64.8(2)	64.9(2.2)	66(2.1)
same-time correlation	39(2.3)	39.6(2.3)	39.6(2.2)	38.9(2.2)	39.7(2.5)	39.3(2.3)	39.1(2.5)
null model	2(0.7)	2.1(0.8)	2(0.7)	2(0.6)	2(0.7)	2.1(0.7)	2(0.6)

Noise strength	1.10η	1.15η	1.20η	1.25η
delayed correlation	66.8(2.1)	66.5(2.7)	66.3(3)	66.4(2.6)
absolute correlation	66.6(1.6)	67.2(2.1)	67.2(1.9)	67.8(1.6)
same-time correlation	39.2(2.4)	38.8(2.5)	38.5(2.4)	38.5(2.9)
null model	1.9(0.7)	2.1(0.8)	2(0.6)	1.9(0.7)

IX. NETWORK RECONSTRUCTION ACCURACY FOR BIOLOGICAL CONNECTOMES

TABLE N. Accuracy of network reconstruction for biological structural networks. Percentage of correctly identified edges when the structural networks are derived from real connectomes (rat, cat, monkey and human) with density from 2% to 28%. The means are followed by standard deviations and calculated over 100 simulations of networks' dynamics by the linear Wilson-Cowan model.

Mouse connectome — **112 nodes, maximum density 52%**

Density	2%	4%	6%	8%	10%	12%	14%
delayed correlation	84.6(2.5)	79.9(2.0)	68.3(1.5)	59.0(1.2)	56.8(1.0)	53.0(0.9)	50.9(0.9)
absolute correlation	45.2(1.5)	45.2(0.7)	40.7(0.8)	38.3(0.8)	40.3(0.8)	39.9(0.8)	40.6(0.8)
same-time correlation	46.1(1.5)	45.4(0.7)	41.2(0.9)	39.1(0.8)	41.9(0.8)	40.8(0.7)	41.6(0.8)
null model	2.7(1.1)	3.9(1.2)	6.0(1.2)	8.2(1.2)	10.1(1.3)	12.2(1.2)	14.1(1.0)

Density	16%	18%	20%	22%	24%	26%	28%
delayed correlation	49.9(0.9)	49.1(0.8)	47.3(0.8)	47.0(0.7)	46.9(0.8)	47.2(0.7)	47.9(0.7)
absolute correlation	41.4(0.8)	42.1(0.7)	41.6(0.7)	42.3(0.8)	42.7(0.7)	43.9(0.8)	45.0(0.7)
same-time correlation	42.3(0.8)	43.1(0.7)	42.6(0.7)	43.4(0.9)	43.8(0.8)	44.8(0.8)	46.1(0.8)
null model	15.9(1.0)	17.9(0.9)	20.0(1.1)	21.8(1.1)	23.8(0.9)	26.0(0.9)	28.0(1.0)

Cat connectome — **95 nodes, maximum density 23.5%**

Density	2.7%	11.5%	23.5%
delayed correlation	88.9(2.9)	62.6(2.1)	65.0(1.8)
absolute correlation	4.8(1.6)	24.0(1.8)	49.1(1.2)
same-time correlation	6.7(2.0)	28.4(1.7)	50.2(1.2)
null model	3.0(1.7)	13.3(1.3)	26.1(1.1)

Macaque connectome — **29 nodes, maximum density 76%**

Density	2%	4%	6%	8%	10%	12%	14%
delayed correlation	91.0(9.2)	85.1(8.2)	91.6(4.8)	94.2(3.4)	94.5(2.5)	86.2(3.0)	75.6(3.4)
absolute correlation	2.0(4.6)	2.3(3.7)	19.2(5.1)	28.9(4.8)	34(4.6)	35.6(4.8)	35.9(4)
same-time correlation	1.1(4)	2.1(3.1)	20.4(4.9)	31.6(4.5)	36.6(4.6)	38.7(4.8)	38.8(3.9)
null model	1.4(3.9)	4.2(5)	6.2(4.3)	8.8(5.1)	9.2(4)	11.2(4.5)	14.2(4.8)

Density	16%	18%	20%	22%	24%	26%	28%
delayed correlation	69.8(3.1)	64.1(2.5)	59.5(3.1)	57.1(3.1)	54.3(3.3)	52.4(2.8)	52.0(2.7)
absolute correlation	37.6(4.3)	38.3(4.1)	38.5(3.3)	39.4(4)	39.7(3.5)	40.2(4)	41.4(3.4)
same-time correlation	40.9(4.4)	42.2(4.2)	42(3.7)	43(3.8)	43.5(3.9)	44.1(3.8)	45.6(3.5)
null model	16(4)	17.3(3.9)	20.5(4.3)	21.8(3.8)	23.6(4.1)	25.6(3.9)	28.1(3.3)

Human connectome — **65 nodes, maximum density 33%**

Density	2%	4%	6%	8%	10%	12%	14%
delayed correlation	96.8(2.5)	96(1.8)	94.7(1.8)	92.8(2)	90.6(1.9)	89.3(2.2)	86.2(2)
absolute correlation	2.1(2.2)	4.5(2)	7.1(2.2)	13.4(2)	19(2.4)	25.4(2.4)	30.5(2.1)
same-time correlation	2.1(2)	5.3(2.4)	8(2.2)	15(2.2)	20.4(2.7)	26.5(2.4)	31(2)
null model	1.7(1.8)	4.1(2.1)	5.9(2.1)	8(2)	9.8(1.8)	11.8(1.7)	13.9(1.9)

Density	16%	18%	20%	22%	24%	26%	28%
delayed correlation	84(2.2)	80.6(2.5)	77.9(2.1)	75.5(1.9)	72.2(2.1)	69.9(1.8)	67.6(1.7)
absolute correlation	34.1(2)	39(1.7)	42.6(1.4)	44.9(1.3)	46.4(1.2)	48(0.9)	49(0.8)
same-time correlation	34.4(2)	39(1.7)	42.7(1.4)	44.9(1.3)	46.4(1.2)	48(0.9)	49(0.8)
null model	16(1.9)	17.9(1.8)	20(1.7)	21.7(1.8)	24.1(1.8)	26.2(1.6)	28.2(1.4)

TABLE O. Accuracy of network reconstruction for biological structural networks. Percentage of correctly identified edges when the structural networks are derived from real connectomes (rat, cat, monkey and human) with density from 2% to 28%. The means are followed by standard deviations and calculated over 100 simulations of networks' dynamics by the linear diffusion model.

Mouse connectome

112 nodes, maximum density 52%

Density	2%	4%	6%	8%	10%	12%	14%
delayed correlation	55.3(4.9)	57.8(3.5)	74.5(2.7)	81.8(1.8)	85.4(1.3)	84.6(1)	83.6(0.8)
absolute correlation	47.3(2)	53(1.3)	59.2(1.3)	59.6(1.2)	62.5(1.2)	61.7(1.2)	62.4(1.1)
same-time correlation	47.2(2)	53(1.3)	59.2(1.3)	59.7(1.3)	62.6(1.2)	61.9(1.1)	62.7(1.1)
null model	2.7(1.1)	3.9(1.2)	6(1.2)	8.2(1.2)	10.1(1.3)	12.2(1.2)	14.1(1)

Density	16%	18%	20%	22%	24%	26%	28%
delayed correlation	80.9(0.9)	77(1.5)	72.6(1.8)	68.5(1.7)	65.8(1.8)	63.2(1.7)	61(1.4)
absolute correlation	61.4(1.2)	60.3(1.1)	59.1(1.1)	58.3(1.1)	58.3(1.1)	58.2(1)	58.3(1)
same-time correlation	61.8(1.2)	60.8(1)	59.5(1.1)	58.8(1.1)	58.9(1)	58.8(1)	58.9(1)
null model	15.9(1)	17.9(0.9)	20(1.1)	21.8(1.1)	23.8(0.9)	26(0.9)	28(1)

Cat connectome

95 nodes, maximum density 23.5%

Density	2.7%	11.5%	23.5%
delayed correlation	47.9(5.3)	86.6(2)	75.6(2)
absolute correlation	35.6(1.5)	50.9(1.4)	49.9(1.4)
same-time correlation	35.6(1.5)	51(1.4)	50.2(1.3)
null model	3(1.3)	13.3(1.3)	26.1(1.1)

Macaque connectome

29 nodes, maximum density 76%

Density	2%	4%	6%	8%	10%	12%	14%
delayed correlation	92.4(9.2)	72.4(9.2)	73.3(9.5)	71.9(8.1)	71.1(6.9)	76.6(6.5)	78.7(5.5)
absolute correlation	10.1(10.3)	2.6(3.6)	27.6(2.7)	42.2(3.1)	45.6(3.2)	54.6(2.5)	55.6(2.4)
same-time correlation	5.9(8.8)	1.3(2.7)	27.6(2.7)	42.2(3.1)	45.6(3.2)	54.6(2.5)	55.6(2.4)
null model	1.4(3.9)	4.2(5)	6.2(4.3)	8.8(5.1)	9.2(4)	11.2(4.5)	14.2(4.8)

Density	16%	18%	20%	22%	24%	26%	28%
delayed correlation	81(3.7)	82.6(3.7)	82.1(3)	83.1(2.6)	81.6(2.2)	81(1.9)	80.3(2)
absolute correlation	60.2(2.7)	61.4(2.5)	60.3(2.4)	62.7(2.8)	61.7(2.7)	63.6(2.3)	65.8(2.7)
same-time correlation	60.2(2.7)	61.4(2.5)	60.3(2.5)	62.9(2.8)	61.8(2.8)	63.8(2.3)	66(2.7)
null model	16(4)	17.3(3.9)	20.5(4.3)	21.8(3.8)	23.6(4.1)	25.6(3.9)	28.1(3.3)

Human connectome

65 nodes, maximum density 33%

Density	2%	4%	6%	8%	10%	12%	14%
delayed correlation	30.3(10.9)	46.9(6.5)	56.9(6.2)	69.2(4.7)	77.8(3.5)	81(3.1)	83.5(2.8)
absolute correlation	2.3(1.2)	3.3(1.5)	4.5(1.5)	9(1.8)	14.2(1.8)	16.5(2.2)	17.6(1.8)
same-time correlation	2.3(1.2)	3.3(1.5)	4.5(1.5)	9.2(1.7)	14.4(1.8)	16.6(2.2)	17.8(1.8)
null model	1.7(1.8)	4.1(2.1)	5.9(2.1)	8(2)	9.8(1.8)	11.8(1.7)	13.9(1.9)

Density	16%	18%	20%	22%	24%	26%	28%
delayed correlation	86.1(2.2)	88.3(1.9)	89.9(1.5)	90.8(1.1)	90.1(1.2)	88.7(1.4)	85.3(1.8)
absolute correlation	19.6(2)	22.1(1.9)	24.5(1.9)	26.8(1.8)	28.4(1.9)	30.2(1.8)	32.4(1.7)
same-time correlation	19.8(2)	22.4(1.8)	24.7(1.8)	27(1.8)	28.7(1.9)	30.5(1.8)	32.7(1.8)
null model	16(1.9)	17.9(1.8)	20(1.7)	21.7(1.8)	24.1(1.8)	26.2(1.6)	28.2(1.4)

TABLE P. Accuracy of network reconstruction for biological structural networks. Percentage of correctly identified edges when the structural networks are derived from real connectomes (rat, cat, monkey and human) with density from 2% to 28%. The means are followed by standard deviations and calculated over 100 simulations of networks' dynamics by the FitzHugh-Nagumo model.

Mouse connectome

112 nodes, maximum density 52%

Density	2%	4%	6%	8%	10%	12%	14%
delayed correlation	36(2.3)	32.6(2.1)	31.2(1.8)	28.3(4)	29.5(3.1)	30(4.7)	33.5(2.7)
absolute correlation	45(1.7)	33.7(1.6)	37.1(1.1)	34(4.9)	31.6(3.4)	32.8(5.2)	36.4(2.8)
same-time correlation	6.3(0.9)	3.7(0.6)	8.7(0.7)	9.8(0.8)	13.4(0.9)	15.5(1.3)	19.6(1.1)
null model	2.8(1.1)	4.2(1.2)	6.1(1.3)	7.8(1.1)	10.2(1.2)	12.1(1.1)	13.9(1)

Density	16%	18%	20%	22%	24%	26%	28%
delayed correlation	34.3(1.3)	36.8(1.2)	37.9(2.8)	39.8(2.9)	42.3(1.5)	44.1(1.4)	45.2(2.9)
absolute correlation	37(0.9)	38.5(1.1)	42.9(3.1)	42.9(3)	44.8(0.9)	46.1(0.9)	46.8(2.9)
same-time correlation	21.2(0.6)	23.9(0.6)	26(1.3)	27.3(1.4)	28.8(0.6)	29.5(0.6)	30.8(1.3)
null model	16(1.1)	18.1(1.1)	20(1.1)	22.1(1)	24(1)	25.8(1)	28.1(0.9)

Cat connectome

95 nodes, maximum density 23.5%

Density	2.7%	11.5%	23.5%
delayed correlation	80.9(2)	58.8(4.7)	41.5(5.4)
absolute correlation	77.7(1.6)	52.2(4.1)	28.7(0.9)
same-time correlation	0.5(0.3)	4.5(1.1)	11.4(5.5)
null model	3(1.3)	13.1(1.3)	26.1(1.1)

Macaque connectome

29 nodes, maximum density 76%

Density	2%	4%	6%	8%	10%	12%	14%
delayed correlation	92.6(9.9)	76.1(12.8)	78.2(6)	74.8(3.1)	72(3.4)	66.7(3.3)	65.5(3.4)
absolute correlation	88.3(3)	81.9(9.3)	72.6(2.7)	72.1(3.4)	73.6(2.4)	69.9(3)	70.4(2.4)
same-time correlation	0(0)	0(0)	8(0)	13.7(1.9)	16.6(2.3)	20(1.8)	20.5(1.6)
null model	3(5.9)	4.2(4.8)	5.3(4.5)	8.1(4.5)	10(4.1)	12.1(4.4)	14.4(4)

Density	16%	18%	20%	22%	24%	26%	28%
delayed correlation	67(2.9)	66.1(3)	62.4(2.6)	60.3(2.7)	57.4(2.2)	56.8(2.7)	55.3(2.7)
absolute correlation	69.8(2.8)	69.4(2.1)	65.5(2.9)	63.6(2.5)	60.6(2.3)	60.6(2.4)	59.2(2.6)
same-time correlation	23.6(1.6) 23.8(1.6)	23.8(1.5)	24.8(1.5)	24.7(1.6)	26.4(1.5)	27.3(1.9)	
null model	16.6(3.7)	17.8(4.3)	19.9(3.9)	22.4(4.3)	24.2(4.2)	26.4(3.7)	27.2(3.2)

Human connectome

65 nodes, maximum density 33%

Density	2%	4%	6%	8%	10%	12%	14%
delayed correlation	59.3(12.3)	60.8(12.5)	66.6(7)	65.9(2.3)	61.2(8.2)	58.4(9.3)	57.8(7.4)
absolute correlation	72(12.7)	70.1(13.8)	69.7(7.1)	66.8(2.1)	59.9(8)	57.3(9.1)	55.5(7.1)
same-time correlation	0.1(0.8)	0.2(0.9)	0.2(0.5)	1(0.7)	2.1(0.9)	3.4(1.1)	5.3(0.8)
null model	1.7(1.9)	4.2(2.5)	5.9(2)	8.1(1.9)	9.9(2)	11.9(1.8)	13.8(2)

Density	16%	18%	20%	22%	24%	26%	28%
delayed correlation	55.8(9.9)	56(5.1)	54(7.8)	52.8(7.1)	51.8(5.1)	50.8(3.4)	50.5(4.1)
absolute correlation	53.8(9.5)	53.9(4.9)	52.4(7.5)	51.6(6.8)	50.7(5)	50.6(3.3)	51.1(4.1)
same-time correlation	5.7(0.7)	6.3(0.5)	6.6(0.9)	7.3(1.2)	7.8(1.5)	8.2(1.4)	9.9(2.1)
null model	16(1.9)	18.1(1.8)	19.9(1.5)	22.1(1.9)	24(1.8)	25.5(1.5)	28.1(1.4)

[1] Petersen S, Sporns O. Brain networks and cognitive architectures. Neuron. 2015;88(1):207–219.
[2] Gallos LK, Makse HA, Sigman M. A small world of weak ties provides optimal global integration of self-similar modules in functional brain networks. Proceedings of the National Academy of Sciences. 2012;109(8):2825–2830.
[3] Messé A, Hütt M, König P, Hilgetag C. A closer look at the apparent correlation of structural and functional connectivity in excitable neural networks. Sci Rep. 2015;5:7870.
[4] Zimmermann J, Ritter P, Shen K, Rothmeier S, Schirner M, McIntosh AR. Structural architecture supports functional organization in the human aging brain at a regionwise and network level. Hum Brain Mapp. 2016;37(7):2645–2661.
